# Amplification-free long-read sequencing reveals unforeseen CRISPR-Cas9 off-target activity

**DOI:** 10.1186/s13059-020-02206-w

**Published:** 2020-12-01

**Authors:** Ida Höijer, Josefin Johansson, Sanna Gudmundsson, Chen-Shan Chin, Ignas Bunikis, Susana Häggqvist, Anastasia Emmanouilidou, Maria Wilbe, Marcel den Hoed, Marie-Louise Bondeson, Lars Feuk, Ulf Gyllensten, Adam Ameur

**Affiliations:** 1 Science for Life Laboratory, Department of Immunology, Genetics and Pathology, Uppsala University, Uppsala, Sweden; 2 Program in Medical and Population Genetics, Broad Institute of Massachusetts Institute of Technology and Harvard, Cambridge, MA USA; 3 Division of Genetics and Genomics, Boston Children’s Hospital, Boston, MA USA; 4 Foundation for Biological Data Science, Belmont, CA USA; 5 The Beijer laboratory and Department of Immunology, Genetics and Pathology, Uppsala University, Uppsala, Sweden; 6 Department of Epidemiology and Preventive Medicine, Monash University, Melbourne, Australia

**Keywords:** CRISPR-Cas9, On-target, Off-target, Long-read sequencing, Single molecule sequencing, PacBio sequencing, Nanopore sequencing, SMRT-OTS, Nano-OTS

## Abstract

**Background:**

One ongoing concern about CRISPR-Cas9 genome editing is that unspecific guide RNA (gRNA) binding may induce off-target mutations. However, accurate prediction of CRISPR-Cas9 off-target activity is challenging. Here, we present SMRT-OTS and Nano-OTS, two novel, amplification-free, long-read sequencing protocols for detection of gRNA-driven digestion of genomic DNA by Cas9 in vitro.

**Results:**

The methods are assessed using the human cell line HEK293, re-sequenced at 18x coverage using highly accurate HiFi SMRT reads. SMRT-OTS and Nano-OTS are first applied to three different gRNAs targeting HEK293 genomic DNA, resulting in a set of 55 high-confidence gRNA cleavage sites identified by both methods. Twenty-five of these sites are not reported by off-target prediction software, either because they contain four or more single nucleotide mismatches or insertion/deletion mismatches, as compared with the human reference. Additional experiments reveal that 85% of Cas9 cleavage sites are also found by other in vitro-based methods and that on- and off-target sites are detectable in gene bodies where short-reads fail to uniquely align. Even though SMRT-OTS and Nano-OTS identify several sites with previously validated off-target editing activity in cells, our own CRISPR-Cas9 editing experiments in human fibroblasts do not give rise to detectable off-target mutations at the in vitro-predicted sites. However, indel and structural variation events are enriched at the on-target sites.

**Conclusions:**

Amplification-free long-read sequencing reveals Cas9 cleavage sites in vitro that would have been difficult to predict using computational tools, including in dark genomic regions inaccessible by short-read sequencing.

## Background

The CRISPR-Cas9 system is one of the most important breakthroughs in modern biotechnology, as it has increased the efficiency and ease of modifying DNA in living cells. CRISPR-Cas9 genome editing in eukaryotic cells was first demonstrated in 2013 [1–4] and has since become an instrumental tool in biomedical research and in bioengineering [5]. CRISPR-Cas9 also shows great promise for clinical use [6], even though the ethical aspects of human germline genome editing require careful consideration [7, 8]. A major reason for caution is that the CRISPR-Cas9 system can induce mutations at locations other than the targeted site [9–11]. Such “off-target” mutations have the potential to disrupt the function or regulation of genes in an unpredictive manner, and consequently, they are a serious concern for CRISPR-Cas9 applications in the medical field [12]. Development of more efficient and precise genome editing tools such as CRISPR-Cas12a [13] or prime-editing [14] could help alleviate some of the off-target concerns. But even with these new tools, off-target mutations cannot be excluded, in particular in cases where the DNA sequence of the cells subjected to genome editing is not completely known.

In any CRISPR-Cas9 genome editing experiment, it is crucial to design a guide RNA (gRNA) that specifically binds to the target of interest, and not to any unintended genomic loci. This gRNA will direct the Cas9 endonuclease to its target, after which Cas9 cleaves the DNA molecule, introducing a double stranded break. The DNA is then repaired either by non-homologous end joining (NHEJ) or by homology-directed repair (HDR). During NHEJ, the DNA is repaired by a ligation process of the Cas9 cleaved ends and small insertion and deletion mutations are typically introduced during this repair step. In the presence of a homologous donor template, usually containing a sequence of interest flanked by homology arms, HDR can be initiated to create a desired mutation through homologous repair [15]. In general, Cas9 cleaves its intended target reliably, but off-target mutations can be introduced if the gRNA also binds to other locations. Another potential side effect of CRISPR-Cas9 editing is that larger structural variations, e.g. insertions and deletions of several hundred base pairs, may be introduced during the DNA-repair process. Such large structural variants (SVs) have been detected at the on-target site [16], but they have not yet been shown to occur at off-target sites. Although there have been conflicting reports on the abundance and consequences of unintended mutations [16–19], there is a consensus that off-target sites should be screened for when designing a gRNA, to increase the chances of a successful and specific genome editing [12].

Guide RNAs are typically designed by computational tools that compare the gRNA sequence to a reference genome and predict the binding affinity both to the on-target sequence as well as to potential off-targets [20–22]. Although intuitively helpful, these tools can yield false-positive or negative results due to the difficulty to exactly model gRNA-DNA binding affinity in an algorithm. Furthermore, the DNA sequence in the cells being investigated can differ substantially from the reference genome used in the computational modeling, potentially resulting in even more false predictions. In recent years, in vitro*-*based assays [23–28] have been developed that allows for experimental detection of Cas9 off-target sites in a particular DNA sample. However, since these methods are based on PCR amplification and short-read sequencing, they have inherent limitations when it comes to detection of Cas9 cleavage in repetitive, low complexity, or AT/GC-rich regions. These issues can be improved by long-read single molecule sequencing technologies. At present, Pacific Biosciences (PacBio) and Oxford Nanopore Technologies (ONT) are the two main providers of long-read sequencing, and it is now widely accepted that these technologies have a superior ability, as compared to short-read sequencing, to resolve SVs as well as other complex regions in the human genome [29–33].

Here we propose two new methods for accurate in vitro detection of gRNA binding and Cas9 cleavage, and we denote these “off-target sequencing” (OTS). The methods are based on PacBio’s single molecule real-time sequencing (SMRT-OTS) and ONT’s nanopore sequencing (Nano-OTS). By introducing these two protocols, rather than just one, we have an alternative amplification-free method to employ for orthogonal validation of our findings. The SMRT-OTS and Nano-OTS methods were evaluated using DNA from the human HEK293 cell line. Importantly, the HEK293 cells were whole genome sequenced to high coverage using long and accurate SMRT sequencing reads, i.e., high-fidelity (HiFi) reads [33], to get the best possible view of the genomic DNA to which the gRNA binds. Finally, we performed CRISPR-Cas9 editing and long amplicon re-sequencing of human fibroblast cells to examine the extent to which our in vitro predicted Cas9 cleavage sites also lead to unintended mutations in living cells.

## Results

### Two new amplification-free protocols for off-target sequencing

**Fig. 1:**
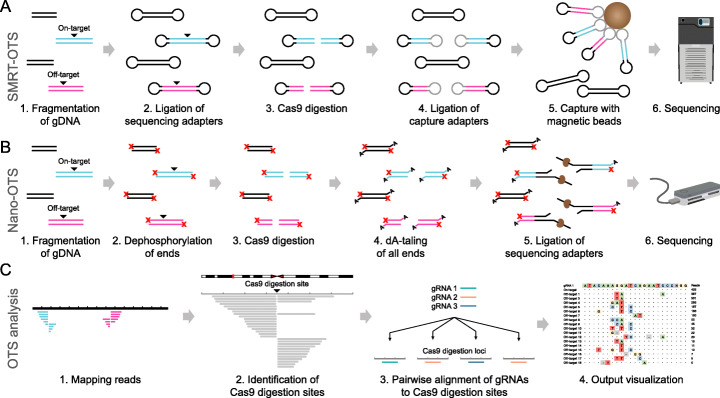
The SMRT-OTS and Nano-OTS methods for Cas9 cleavage detection. **a** SMRT-OTS starts from a DNA sample that has been fragmented by random shearing. The DNA fragments will either contain an on-target site (blue), an off-target (pink), or no gRNA binding site (black) (1). After fragmentation, a SMRTbell library is prepared by ligating sequencing adapters to both ends (2). The SMRTbells containing gRNA binding sites are then cleaved by Cas9 (3), after which capture adapters (gray) are ligated to the cleaved molecules (4). Finally, magnetic beads are used to capture SMRTbells containing the capture adapter (5). This gives an enrichment of SMRTbells cleaved by Cas9 and the enriched library is sequenced on the PacBio Sequel system (6). **b** Nano-OTS, just like SMRT-OTS, starts from a randomly fragmented DNA sample (1). All ends of the fragmented DNA molecules are dephosphorylated to block adapter ligation (2). The DNA fragments containing gRNA binding sites are cleaved by Cas9 (3) and all 3′ ends are dA-tailed (4). Sequencing adapters are then ligated to Cas9 cleaved and dA-tailed ends (5) and these molecules are sequenced on the ONT MinION system (6). **c** The same computational approach is used both for SMRT-OTS and Nano-OTS. Reads are aligned to a reference genome, which gives rise to specific patterns at Cas9 cleavage sites with multiple reads starting at the same position (1). The in-house developed software Insider searches for such patterns in the alignment file and reports all detected targets in a bed file (2). The reference sequence is extracted for all the targets and aligned to the gRNA sequences. Only regions with sufficient similarity to a gRNA sequence are kept (3). The results are reported in an output file that contains the Cas9 cleavage position, the sequence alignment of the gRNA to the reference, and the peak height from the OTS sequencing (4)

We developed two methods for gRNA off-target sequencing (OTS) (Fig. 1a, b). SMRT-OTS is based on PacBio’s SMRT sequencing and produces highly accurate circular consensus sequencing (CCS) reads, which can be used to detect the exact Cas9 cleavage sites as well as genetic variants in on- and off-target regions. Nano-OTS is based on ONT’s nanopore sequencing and allows for rapid identification of Cas9 cleavage sites but with lower per-read accuracy. Our methods are inspired by previously proposed assays where a single gRNA was used to perform Cas9 target enrichment. SMRT-OTS is a modified version of a protocol we previously applied for detection of repeat expansions in human cell lines and blood samples [34, 35], while Nano-OTS is adapted from a targeted sequencing assay [36] used for detection of unknown fusion gene partners [37]. In addition to wet lab assays, we developed a computational method that can be used to identify Cas9 cleavage sites at single base pair resolution, both from high-quality SMRT reads and from lower quality nanopore reads (Fig. 1c). In the analysis, candidate Cas9 cleavage sites are found from specific patterns in the alignment where several reads start or end at the exact same position. Because the reads from our OTS assays originate from randomly sheared DNA fragments with varying start and end positions, such patterns are highly unlikely to arise from background reads that have not been cleaved by Cas9. For multiplexed runs with several gRNAs, pairwise alignments are performed between gRNA sequences and predicted Cas9 cleavage regions to determine which gRNA is bound to each target. Optionally, peaks with little or no resemblance to any gRNA sequence can be removed. See the “Methods” section for more details on the analysis procedure and parameters in this study.

### Detection of Cas9 cleavage sites in human DNA using SMRT-OTS

**Fig. 2:**
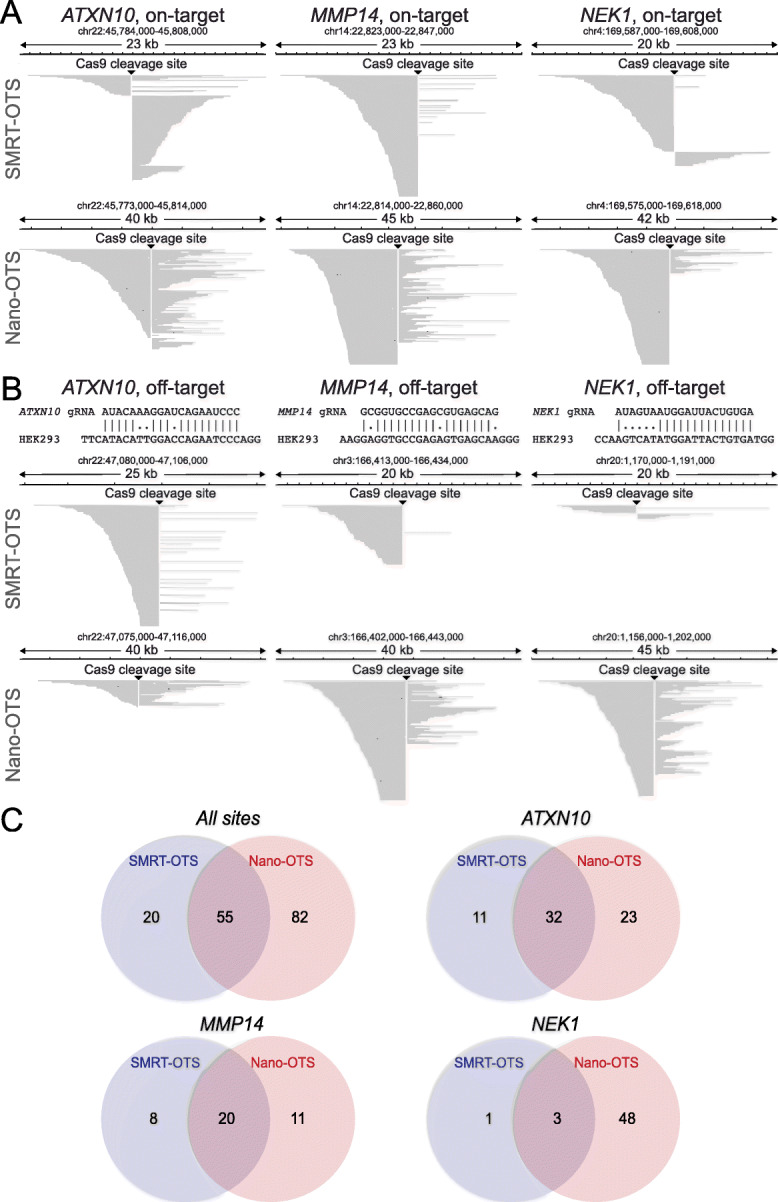
Comparison of results from SMRT-OTS and Nano-OTS. **a** IGV alignments [38] showing the read distributions for SMRT-OTS (top) and Nano-OTS (bottom) in 20–50-kb windows spanning the *ATXN10*, *MMP14*, and *NEK1* on-target sites. The Cas9 cleavage sites are clearly visible as sharp vertical lines where the alignments start or end. Usually, most of the reads are found either upstream or downstream of the Cas9 cleavage site and this imbalance is due to the orientation of the gRNA-Cas9 complex. Adapter ligation is less efficient on the 5′-side of the gRNA where the enzyme remains bound to the DNA after Cas9 cleavage [36]. **b** Examples of off-target alignment peaks for *ATXN10* (left), *MMP14* (middle), and *NEK1* (right). At the top are sequence alignments between the gRNA sequence and the HEK293 genome at the off-target site. There are three single nucleotide mismatches at the *ATXN10* and *MMP14* off-target sites, and five single nucleotide mismatches at the *NEK1* off-target site. All three off-target sites are visible in both in the SMRT-OTS and Nano-OTS alignment data. **c** Venn diagrams showing the overlap between gRNA predictions for SMRT-OTS and Nano-OTS for all three gRNAs combined (leftmost diagram) and each individual gRNA (the three other diagrams)

DNA from the human embryonic kidney cell line HEK293 was used to evaluate the OTS protocols. As a baseline for our experiments, a comprehensive genome map of the HEK293 cells was generated by HiFi SMRT sequencing [33], resulting in 18x whole genome coverage with >Q20 reads of an average read length of 15 kb (Additional file 1: Figure S1). Because of their length and accuracy, the HiFi reads are ideal both for detection of single nucleotide variants (SNVs) and larger SVs [33]. After having determined the HEK293 genome sequence, we performed a multiplexed SMRT-OTS run with three guide RNAs, designed to target an intron of *ATXN10*, and early exons of *MMP14* and *NEK1*. These three gRNAs have all been used in previous experiments, by us and others (see the “Methods” section; Additional file 1: Table S1). Sequencing was performed on a Sequel 1 M SMRT cell, resulting in a total of 57,644 reads with an average read length of 4.0 kb. All three gRNA on-target sites were successfully detected, along with 42 off-targets for *ATXN10*, 27 off-targets for *MMP14*, and three off-targets for *NEK1* (Additional file 2). The on-target alignment peaks for the three gRNAs, as well as examples of off-target peaks with at least three mismatches to the HEK293 genome, are shown in Fig. 2a, b. Throughout the text, we use the term OTS-sites to denote on- and off-target sites detected by our methods.

### Validation of Cas9 cleavage sites using Nano-OTS

To validate our results and to examine the reproducibility of our sequencing protocols, we performed a Nano-OTS run using the same HEK293 DNA and the same three gRNAs. A total of 185,145 reads of average length 7.5 kb were generated using one MinION flow cell. Fifty-four, 30, and 50 OTS-sites were found for *ATXN10*, *MMP14*, and *NEK1*, respectively (Additional file 3). Due to the nature of nanopore sequencing and its primary analysis, the OTS-sites are sometimes predicted within a 10–20-bp interval instead of at exact base pair resolution. Fifty-five OTS-sites overlapped between the two methods, while 20 were found only by SMRT-OTS and 82 only by Nano-OTS (Fig. 2c; Additional file 4). We next performed random sampling of the Nano-OTS data to obtain the same number of reads as for SMRT-OTS (Additional file 5). A total of 66 sites were detected in the downsampled Nano-OTS data, and 33 (50%) of these sites were found by SMRT-OTS (Additional file 1: Figure S2). Although the percentage of overlapping sites was higher for the downsampled data as compared to the original analysis, our results suggests that the differences in coverage only can explain a small part of the differences between the two methods and that there might also be other differences, for example in Cas9 cleavage efficiency.

### Guide RNAs may induce Cas9 cleavage despite high-sequence dissimilarity

**Fig. 3:**
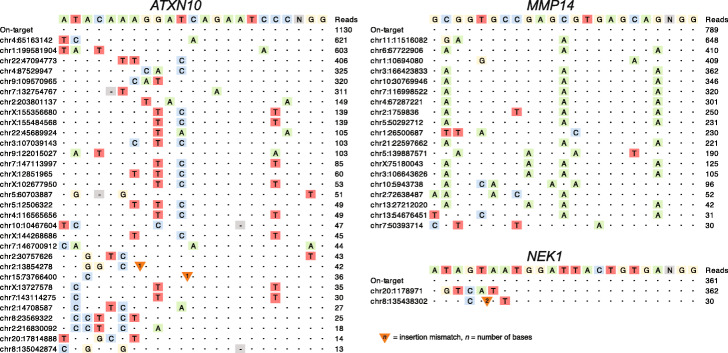
High confidence OTS-sites in HEK293 cells. Visualization of the 55 gRNA binding sites for *ATXN10*, *MMP14*, and *NEK1* that were detected both by SMRT-OTS and Nano-OTS. The letters at the top show the gRNA sequence and the bases (NGG) that corresponds to the PAM site. Below are the on-target and all off-target sequences detected by the OTS methods. Colored letters correspond to single nucleotide mismatches in the HEK293 genome as compared with the gRNA sequence. Triangles and hyphens (-) are used to mark sites where nucleotides need to be inserted or deleted, respectively, in the HEK293 genome in order to match the gRNA sequence. The column to the right contains the combined read count from the SMRT-OTS and Nano-OTS assays, for each OTS-site

As SMRT-OTS and Nano-OTS are two orthogonal methods, we considered the intersection of their OTS-sites (*n* = 55) to be a high-confidence set of targets predicted to be cleaved by Cas9 in the HEK293 cells, and this dataset is visualized in Fig. 3. For comparison, we used the latest release of CHOPCHOP [22] to predict Cas9 cleavage in silico. A total of 82 sites were reported by CHOPCHOP when allowing for up to three single nucleotide mismatches (Additional file 6). Of these in silico predictions, as many as 45 (55%) were not detected by SMRT-OTS or Nano-OTS. These could either be sequences not bound by a gRNA despite high similarity, sites bound by a gRNA but not cleaved by Cas9, or a combination of both. Conversely, 25 (45%) of our OTS-sites were not reported by CHOPCHOP (Additional file 1: Table S2). Among these, 18 OTS-sites had at least four single nucleotide mismatches to the gRNA sequence, and seven OTS-sites contained insertion/deletion mismatches to the gRNA sequence. Three of the OTS-sites have a mismatch in the PAM sequence (NGG), but for all those cases, a canonical PAM sequence can be found at the subsequent position.

### Comparing OTS to other in vitro methods for Cas9 cleavage detection

**Fig. 4:**
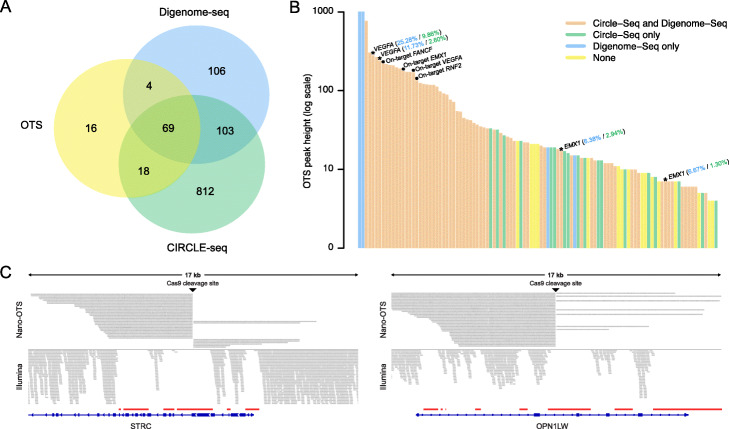
Comparison of OTS to other in vitro-based methods. **a** Venn diagram showing the overlap in results between OTS, Digenome-seq, and CIRCLE-seq for the four gRNAs *EMX1*, *FANCF*, *RNF2*, and *VEGFA*. OTS identified a total 107 sites for the four gRNAs. All 107 sites were detected by Nano-OTS and a subset (*n* = 26) of these were found also by SMRT-OTS. In previous in vitro experiments using the same four gRNAs, CIRCLE-seq and Digenome-seq identified 1002 and 282 binding sites, respectively. **b** Barplot showing the combined SMRT-OTS and Nano-OTS signals for the 107 sites detected by the off-target sequencing methods. The signals are shown on a log10 scale and the bars have been sorted in descending order. Bars are colored to indicate Cas9 cleavage sites detected by Digenome-seq (blue), CIRCLE-seq (green), or by both methods (orange). Yellow bars correspond to sites not detected by Digenome-seq or CIRCLE-seq. On-target sites for *EMX1*, *FANCF*, *RNF2*, and *VEGFA* have been marked by solid black circles. The asterisks mark off-target sites for *EMX1* and *VEGFA* where editing was detected in cells in two previous CRISPR-Cas9 experiments: the Digenome-seq study [25] and the CIRCLE-seq study [28]. The blue and green numbers correspond to the fraction of edited molecules from the Digenome-seq and CIRCLE-seq study, respectively. **c** Two examples of “dark” genomic regions, *STRC* and *OPN1LW*, where Nano-OTS successfully identified an on-target site while short-read data failed to uniquely align. The Nano-OTS reads are displayed at the top and at the bottom is data from one individual from the SweGen dataset [39], sequenced to 30x coverage using Illumina paired-end 150 bp reads. The red lines mark the coordinates for dark genomic regions reported from the study by Ebbert et al. [32]

To compare the performance of our off-target sequencing methods to other in vitro approaches for Cas9 cleavage detection, we performed SMRT-OTS and Nano-OTS with four gRNAs targeting the *EMX1*, *FANCF*, *RNF2*, and *VEGFA* genes (Additional file 7). These gRNAs have been used for detection of off-target sites in Digenome-seq [25] and CIRCLE-seq [28]. Nano-OTS identified 107 sites in total for the four gRNAs, and SMRT-OTS detected a subset of these (*n* = 26) but no additional sites. Of the 107 OTS-sites, 91 (85%) were previously reported by Digenome-seq or CIRCLE-Seq (Fig. 4a). In addition, a large number of Cas9 cleavage sites were reported by Digenome-seq (*n* = 209) and CIRCLE-seq (*n* = 915), while not detected by our OTS methods. As shown in Fig. 4b, all OTS-sites with higher signals (OTS peak height > 23) were reported either by CIRCLE-seq or Digenome-seq. At four of the *EMX1* and *VEGFA* OTS-sites, off-target editing activity was validated in human cells both in the CIRCLE-seq [28] and Digenome-seq study [25], at levels ranging between 1 and 25% (Additional file 1: Table S3). These four edited sites were all identified by Nano-OTS, while SMRT-OTS detected the two *VEGFA* off-targets but missed the two weaker *EMX1* off-targets. The SITE-seq method [23] has been applied to *FANCF* and *VEGFA* in a series of experiments with varying ribonucleoprotein (RNP) concentrations in a range from 0.25 to 1024 nM. All of the 25 OTS-sites for *FANCF* and 60/61 OTS-sites for *VEGFA* were identified in the SITE-seq runs at 64 nM (RNP) concentration (Additional file 1: Figure S3). However, SITE-seq detected many more Cas9 cleavage sites than OTS at concentrations of 64 nM and above, and the largest reciprocal overlap between the two methods was detected at 4–16 nM concentration.

We also performed a separate Nano-OTS run where the Cas9 digestion step was performed using single gRNAs, instead of a pool of gRNAs. This single-plex run resulted in 75 OTS-sites for *EMX1*, *FANCF*, *RNF2*, and *VEGFA* (Additional file 8), and of these, 60 (80%) were detected also in the previous run (Additional file 1: Figure S4). Since more sites were found in the multiplexed run than in the single-plex, we are confident that low-level multiplexing does not have a major negative impact on the sensitivity.

### OTS can detect Cas9 cleavage sites in “dark” regions of the human genome

We hypothesized that our amplification-free long-read sequencing methods would enable detection of Cas9 cleavage activity in complex and repetitive genomic regions. To investigate this further, we designed gRNAs in six “dark” genic regions of the human genome: *CRYAA*, *HSPA1A*, *IKBKG*, *OPN1LW*, *OTOA*, and *STRC* (Additional file 1: Table S1). These six gene targets were selected from a recent study by Ebbert et al. [32], where the authors identified 36,794 dark regions within 6054 disease-relevant gene bodies, where standard whole-genome Illumina data failed to uniquely align. We further examined a previously generated 30x Illumina WGS dataset (SweGen) [39] and could verify that the six target sites lacked coverage in short-read data. In contrast, the reads from a multiplexed Nano-OTS run could be uniquely aligned to the target regions, resulting in successful identification of all six Cas9 cleavage sites (Fig. 4c; Additional file 1: Figure S5). Nano-OTS also detected 24 off-target sites, and 7 (29%) of these were overlapping with a dark genomic region (Additional file 9; Additional file 1: Table S4). Our results thus confirm that long reads enable detection of Cas9 on- and off-target activity in regions difficult to study with short reads.

Human long-read assemblies have been shown to contain several megabases (Mbs) of novel sequences or alternative haplotypes with high diversity from the GRCh38 reference [40, 41]. To determine whether any additional off-targets could be detected in such “novel” regions of the HEK293 genome, a de novo assembly of the HEK293 HiFi data was performed using Peregrine [42], resulting in a genome size of 2896 Mb with N50 of 11.2 Mb (Additional file 1: Table S5). We next aligned the SMRT-OTS reads to the HEK293 de novo assembly and could identify 43, 27, and four OTS-sites for *ATXN10*, *MMP14*, and *NEK*, respectively (Additional file 10). While we were not able to detect any new sites in this way, 74 of the 75 (98.7%) of gRNA binding sites found in GRCh38 could be identified.

### A single nucleotide polymorphism can induce allele-specific Cas9 cleavage

**Fig. 5:**
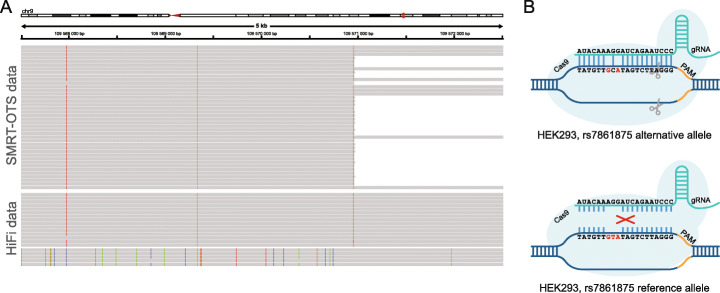
Genetic variation in HEK293 induces allele-specific Cas9 cleavage. **a** An IGV image showing SMRT-OTS alignments (top) and HEK293 HiFi SMRT sequencing reads (bottom) in a window surrounding a predicted gRNA site for *ATXN10* (chr9:109,570,956; GRCh38 coordinates). The HiFi reads reveal a heterozygous SNV (rs7861875; T>C) in HEK293 within the *ATXN10* gRNA binding site, where the reference allele (T) is linked to the alternative alleles of several other heterozygous SNVs in the window. In the SMRT-OTS data, 101 of the 106 reads (95%) of the reads correspond to the haplotype having the alternative rs7861875 allele (G). **b** Schematic view of allele specific *ATXN10* gRNA binding at the rs7861875 locus. The CRISPR-Cas9 complex does not bind to the rs7861875 reference allele (T), which has three mismatches to the gRNA sequence (bottom). It binds more efficiently to the alternative allele (**c**) which has only two mismatches to the *ATXN10* gRNA sequence (top)

The HiFi data for HEK293 allowed us to identify and phase genetic variants across the entire genome. Based on this information, we could investigate allelic biases in Cas9 cleavage for all seven SMRT-OTS datasets. One allele-specific digestion event was found, at an off-target site for *ATXN10*. At this site, HEK293 was reported heterozygous for the T/C SNV rs7861875 (Fig. 5a). The HiFi data further revealed a haplotype with several additional SNVs in the region, all of them linked to the reference allele of rs7861875 (T). The rs7861875 T allele and associated SNV haplotype is present in six of 23 HiFi reads (26%), and the deviation from 50% as well as elevated coverage in the HiFi data suggests that this locus may be duplicated in HEK293 cells even though SV calling failed to report such events (Additional file 1: Figure S6; Additional file 11). In the SMRT-OTS data, 101 of the 106 reads (95%) contain the alternative allele, and only five reads (5%) carry the T allele and associated SNV haplotype. This is consistent with a preferential gRNA binding to the C allele, which has higher sequence similarity (two mismatches) to the *ATXN10* gRNA as compared with the T allele (three mismatches) (Fig. 5b). Although this is just one example, it demonstrates that common genetic variation can cause unintended Cas9 digestion and that our methods are sensitive enough to identify such events. Only one additional heterozygous SNV was present in an OTS-site (*MMP14*; chr2:1759836), but the SMRT-OTS coverage in that region was too low to study allele specific binding.

### Studying in vivo CRISPR-Cas9 off-target effects in human cells

**Fig. 6:**
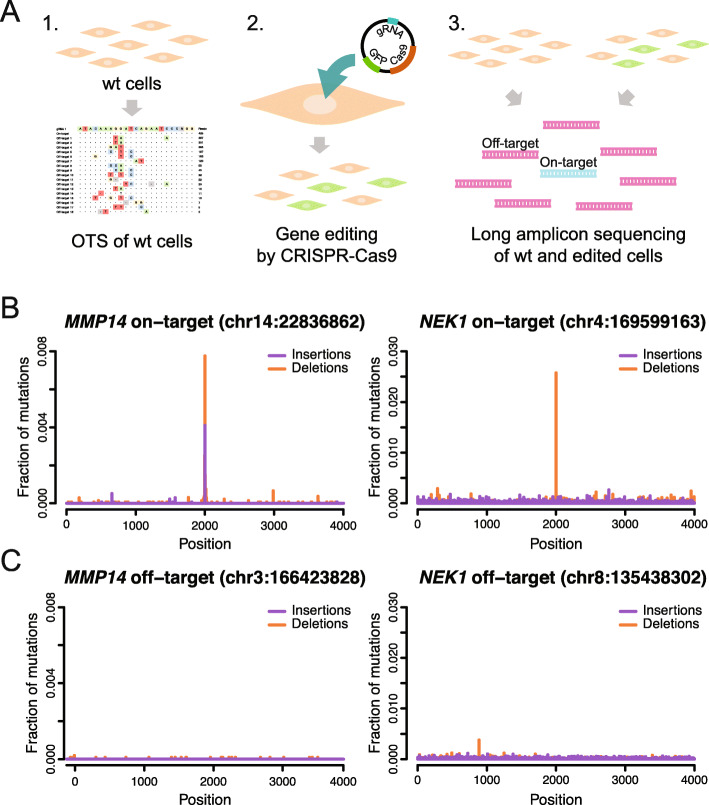
Analysis of on- and off-target editing in CRISPR-Cas9 edited human cells. **a** Overview of experiment to examine genome editing at on- and off-target sites in cells. In the first step, OTS is performed on wild-type (wt) DNA to identify potential off-target sites for a specific gRNA. Secondly, CRISPR-Cas9 genome editing is performed using the same gRNA to generate a population of cells where some of them have been successfully edited (in green). In the third and final step, PCR primers are designed at the on- and off-target sites and long amplicon SMRT sequencing is performed both in the wt DNA as well as in the edited DNA. By analyzing and comparing the resulting amplicon reads for edited and wild-type DNA, it is possible to determine whether unintended genome editing occurs both at the on-target site as well as at the off-target sites. **b** The plots show how insertions and deletion mutations are distributed in a 4-kb window surrounding the Cas9 on-target cleavage sites for *MMP14* (left) and *NEK1* (right). In each plot, the *y*-axis shows the percentages of reads from the edited fibroblast cells that contain a start position for an indel, subtracted by the same indel percentages from the wild-type fibroblast cells. In this way, the background distribution of indel mutations is corrected for. **c** Similar plots as above, but instead for an off-target site for *MMP14* (left) and *NEK1* (right). No overrepresentation of insertion or deletion mutations are found at the off-target Cas9 cleavage sites

The fact that CRISPR-Cas9 cleaves DNA at a specific location in vitro does not necessarily imply that mutations are induced in living cells. Even though the results in Fig. 4b confirmed the presence of off-target mutations for *EMX1* and *VEGFA* in edited cancer cell lines (Additional file 1: Table S3), we designed an experiment on human primary dermal fibroblasts to investigate off-target effects in cells with a normal karyotype (Fig. 6a). Independent CRISPR-Cas9 genome editing experiments of the fibroblast cells were performed using *MMP14* and *NEK1* gRNAs. About 10–15% of the cells were successfully transfected in both experiments, and DNA was extracted from the whole cell population obtained after genome editing, with no additional culturing of individual clones. This implies that only a low fraction of cells (at most 15%) are expected to be edited after the CRISPR-Cas9 experiment. The *MMP14* and *NEK1* on-target sites as well as 19 of the detected off-target sites were then investigated using long amplicon re-sequencing, both in DNA from CRISPR-Cas9 edited fibroblasts as well as from unedited fibroblasts. As expected, the edited cells show an enrichment of indel mutations occurring in proximity to the *MMP14* and *NEK1* on-target sites (Fig. 6b), and the estimated on-target editing efficiency was 32–48% for *MMP14* and 58–87% for *NEK1*. In agreement with results from a recent study by Kosicki et al. [16], several large insertions and deletions (> 50 bp) were detected at the on-target sites using the software SVIM [43] (Additional file 1: Table S6). Interestingly, all large insertions have high similarity to the CRISPR-Cas9 genome editing vector (Additional file 12) and likely were incorporated in the DNA repair process. However, none of the 19 investigated off-target sites showed an enrichment of indel mutations in proximity to the Cas9 cleavage site (Fig. 6c; Additional file 1: Tables S7-S8). Our results thus show that CRISPR-Cas9 genome editing occurred at the on-target sites, but not at the off-target sites, in this experiment on human fibroblast cells.

## Discussion

Amplification-free long-read sequencing technologies can access repetitive and extreme GC-regions of the genome in an unbiased manner [33, 44]. Therefore, the SMRT-OTS and Nano-OTS methods have a considerable advantage when it comes to detection of Cas9 cleavage in “dark” regions of the genome, where the short reads used in other in vitro-based assays fail to uniquely align. Since dark regions have been found in over 6000 gene bodies in the human genome [32], many of which are of known medical relevance, it could be of great importance to correctly determine on- and off-target Cas9 cleavage sites in such loci when performing genome editing experiments.

In this study, four gRNAs were multiplexed on PacBio’s Sequel system and six gRNAs on ONT’s MinION instrument, but it should be possible to increase the degree of multiplexing by an order of magnitude using the higher throughput Sequel II and PromethION systems. Higher order multiplexing could be useful when screening large gRNA panels for optimal candidates in gene knockout experiments, or for post-hoc quantification of off-target effects. Although both OTS protocols are based on single molecule long-read sequencing, each method has its own unique features. SMRT-OTS has the advantage of producing high-quality CCS reads, thereby enabling accurate SNV calling in the molecules cleaved by Cas9. Nano-OTS, on the other hand, is a very fast protocol (< 1 day) that utilizes the portable and easily accessible MinION sequencer. When it comes to DNA input amount, the requirements for SMRT-OTS (~ 10–15 μg) and Nano-OTS (~ 5–10 μg) are similar to what is used for SITE-seq [23] and Digenome-seq [25], despite that no amplification is performed in the OTS protocols. CIRCLE-seq requires substantially larger amounts of input DNA (25 μg) [28].

Eighty-five percent of the OTS-sites for *EMX1*, *FANCF*, *RNF2*, and *VEGFA* were found also by Digenome-seq and CIRCLE-seq, suggesting a low fraction of false positives in the OTS results. However, a substantial number of sites detected by Digenome-seq (*n* = 209) and CIRCLE-seq (*n* = 915) were not found by OTS. There are several possible explanations for these discrepancies, such as the higher sequencing throughput in Illumina-based methods, differences in concentrations of Cas9, gRNAs and genomic DNA, or other experimental differences between the assays. There might also be a fraction of false-positive Cas9 cleavage sites among those detected by only one of the methods. The comparison to SITE-seq shows that gRNA and Cas9 concentrations are important factors that have a big influence on the number of Cas9 cleavage sites detected in the in vitro experiments. Since the OTS results have highest concordance to SITE-seq results with moderate RNP concentrations (4–16 nM), this indicates that gRNA/Cas9 concentration have not been saturated in our experiments. Using higher concentrations would likely allow us to identify more Cas9 cleavage sites, including weaker sites only bound in high gRNA/Cas9 concentrations. In this study, we have not performed any direct comparisons to cell-based methods like GUIDE-seq [45] or DISCOVER-seq [46]. Such cell-based experiments can be very informative, in particular since they determine Cas9 cleavage in living cells, but in vitro-based assays like OTS have the advantages of being faster, simpler, and not requiring any genome editing experiments.

A unique aspect of our gRNA binding experiment is that we determined the exact genetic background of the HEK293 cells. For this, we used state-of-the-art HiFi whole-genome sequencing [33]. The HEK293 HiFi data, coupled with results from the OTS-assays, gives us a more detailed view of gRNA on- and off-target activity in human DNA than ever before. In fact, we were able to detect a vast majority of the gRNA binding sites without making use of the human GRCh38 reference, by instead using the de novo assembled HEK293 genome for the OTS analyses. One intriguing finding was preferential binding of the *ATXN10* gRNA to the alternative allele at rs7861875. Although one should be careful to draw general conclusions from a single example observed in vitro, this result suggests that SNVs can induce unexpected off-target activity and that individual level genetic variation should be taken into consideration when designing gRNAs for medical purposes. Computational strategies that take into account SNVs in Cas9 cleavage prediction already exist [47, 48], but those rely on databases that do not contain all variants that any individual carries. Our results further demonstrate that gRNAs can induce Cas9 digestion in genomic DNA despite having three or more single nucleotide mismatches, or even insertion or deletion mismatches. Since cleavage sites with high-sequence divergence are difficult to predict using computational tools, we argue that in vitro tools like the ones presented here are needed to accurately determine where a gRNA induces unintended Cas9 cleavage in a particular DNA sample.

The results in Fig. 4b confirm that the OTS methods can find Cas9 cleavage events in vitro that lead to off-target genome editing in cells. However, our CRISPR-Cas9 experiments in fibroblast cells showed no evidence of genome editing at any of the off-targets, even though editing was clearly detected at the *MMP14* and *NEK1* on-target sites. We can only speculate about these results, but it might be the case that gRNA and Cas9 concentrations were higher in the OTS experiments as compared to in our genome editing experiments, thereby forcing the Cas9 to cleave at off-target sites that are not affected in living cells where the concentrations of CRISPR-Cas9 components are lower. In support of this hypothesis, it has previously been observed that off-target genome editing can be increased by prolonging expression of RNP, altering the delivery method, and/or changing cell type [23]. It might be the case that chromatin structure, DNA repair systems, or other mechanisms in the fibroblast cells prevent the CRISPR-Cas9 system to induce off-target mutations. Although our results in fibroblast cells agree with previous studies where off-targets failed to be detected in living cells [19, 49–51], other studies have confirmed off-target editing both in cells and organisms [28, 45, 46]. Therefore, it is necessary to be cautious and to independently examine each gRNA and each cell type for potential off-target effects.

## Conclusions

In summary, with SMRT-OTS and Nano-OTS, we provide new tools to evaluate and improve gRNA design, as well as to optimize CRISPR protocols. Coupled with high accuracy long-read whole genome sequencing, we believe these methods will enable us to better understand the mechanisms of gRNA binding and, hopefully, also to prevent negative effects of off-target and unintended mutations in future CRISPR-Cas9 experiments.

## Methods

### Samples

Genomic DNA from the HEK293 cell line was purchased from GenScript. Human primary dermal fibroblasts were purchased from ATCC. The cell lines have not been authenticated.

### Whole-genome HiFi SMRT sequencing of HEK293 on Sequel II

To generate a HiFi library, genomic DNA was sheared using the Megaruptor 2 (Diagenode) with a long hydropore and a 20-kb shearing protocol. Size distribution of the sheared DNA was characterized on the Femto Pulse system (Agilent Technologies) using the Genomic DNA 165 kb Kit. Sequencing libraries were constructed using the protocol “Preparing HiFi SMRTbell Libraries using SMRTbell Express Template Prep Kit 2.0” from PacBio. SMRTbells were size selected using 0.75% agarose 1–18 kb protocol on SageELF (Sage Science) according to the HiFi SMRTbell library protocol. Size-selected SMRTbells were examined on the Femto Pulse system (Agilent Technologies) using the Genomic DNA 165-kb Kit. Library fraction of 15 kb and 17 kb was selected for sequencing. Sequencing was performed on two SMRT cells using the Sequel II system and the 2.0 sequencing and binding chemistry, with 2 h pre-extension and 30 h movie time.

### Guide RNAs

The gRNAs used in this study have were purchased from Integrated DNA Technologies and their sequences and genomic location is available in Additional file 1: Table S1. The *ATXN10* gRNA was used in our previous experiments on amplification-free PacBio sequencing of repeat expansions in the human genome [34, 35]. The *MMP14* gRNA has been used by us and others in genome editing experiments. The *NEK1* gRNA has been used in genome editing experiments by Horizon Discovery, and a *NEK1* edited HAP1 cell line can be ordered from their website (https://horizondiscovery.com). The *EMX1*, *FANCF*, *RNF2*, and *VEGFA* gRNAs have been used in previous publications [23–25, 28] for studying CRISPR-Cas9 off-target effects. The *CRYAA*, *HSPA1A*, *IKBKG*, *OPN1LW*, *OTOA*, and *STRC* gRNAs were designed to target dark genic regions [32] using the CHOPCHOP gRNA design tool [22].

### SMRT-OTS: off-target sequencing using PacBio’s SMRT sequencing

SMRT-OTS libraries were prepared in a similar manner described by Tsai et al. [34], with modifications. Genomic DNA was sheared to 8-kb fragments using Megaruptor 2 (Diagenode). Standard SMRTbell libraries were prepared using Template Preparation Kit 1.0 (Pacific Biosciences) according to the manufacturer’s instructions. An extra exonuclease treatment, using Exonuclease I (New England Biolabs) and Lambda exonuclease (New England Biolabs), was added at the end of the library preparation. The final SMRTbell library was size selected using the Blue Pippin system (Sage Science) with a cut-off at 4 kb. The crRNA and tracrRNA with Alt-R modification (Integrated DNA Technologies) were annealed in a 1:1 ratio to form gRNA that was used in the Cas9 (New England Biolabs) digestion of the SMRTbell libraries. Cas9 and gRNA in the presence of buffer were incubated at 37 °C for 10 min, before heparin was added and the mixture was incubated for an additional 3 min at 37 °C. One microgram of SMRTbell library was then added and incubated for 1 h at 37 °C. EDTA was added to terminate the reaction and the SMRTbell library was subjected to PB AMPure bead (Pacific Biosciences) purification. Hairpinned capture adapters with a polyA-stretch (5′-ATCTCTCTCTTAAAAAAAAAAAAAAAAAAAAAAATTGAGAGAGAT-3′) were ligated, overnight at 16 °C, to the Cas9 digested SMRTbell molecules using T4 DNA ligase (Thermo Fischer Scientific) forming asymmetrical SMRTbell libraries. The asymmetrical SMRTbell library was subjected to exonuclease III and VII (Pacific Biosciences) at 37 °C for 1 h. MagBeads (Pacific Biosciences) were used to enrich for asymmetric SMRTbell molecules by binding to the capture hairpin-adapters. The asymmetric SMRTbell molecules/MagBead complex was incubated under rotation at 4 °C for 2 h in MagBead Binding buffer v2 (Pacific Biosciences) three times. Finally, the enriched asymmetric SMRTbells were eluted in Elution buffer (Pacific Biosciences) for 10 min at 50 °C. The asymmetric SMRTbell molecules were prepared for SMRT sequencing by primer annealing with standard PacBio sequencing primer lacking the polyA sequence for 1 h at 20 °C. Sequel DNA polymerase 3.0 was bound to the template/primer complex for 4 h at 30 °C. Sequencing was performed on the PacBio Sequel system using one 1 M SMRT cell, Sequel Sequencing kit 3.0, and a 600-min movie time. Asymmetric SMRTbell template sequencing data was subjected to a customized analysis pipeline for capture and conventional hairpin-adapter recognition for separating subreads. Subsequently, the CCS tool in SMRT analysis was used to create circular consensus sequencing reads from the subreads. A detailed step-by-step instruction of the SMRT-OTS protocol is available on protocols.io (https://www.protocols.io/view/smrt-ots-bjugkntw) [52].

### Nano-OTS: off-target sequencing using ONT’s nanopore sequencing

Genomic DNA was sheared to 20-kb fragments using Megaruptor 2 (Diagenode) and size selected using the BluePippin system (Sage Science) with a cut-off at 10 kb. Three to 4 μg of sheared and size-selected DNA was prepared using the Cas9-mediated PCR-free protocol provided by Oxford Nanopore technologies with minor modifications. The crRNA and tracrRNA with Alt-R modification (Integrated DNA Technologies) were annealed in Duplex buffer (Integrated DNA Technologies) at 95 °C for min and were then allowed to cool down to room temperature. Ribonucleoproteins (RNPs) were formed by combining the annealed gRNA, HiFi Cas9 (Integrated DNA Technologies) and 1x NEB CutSmart buffer (New England Biolabs) and incubated at room temperature for 30 min. The fragmented and size-selected DNA was dephosphorylated to block all ends from ligation of adapters in a downstream adapter ligation step. Subsequently, the DNA molecules were digested by Cas9 using the previously prepared RNPs and the newly cleaved ends were dA-tailed to enable adapter ligation. The library preparation was completed by ligation of adapters from the SQK-LSK109 kit (Oxford Nanopore Technologies) and cleaned up with AMPure XP beads (Beckman Coulter) before preparation for sequencing. Sequencing was performed using the MinION system (Oxford Nanopore Technologies) with a R9.4.1 flow cell and Guppy v3.3.3 was used for base calling. A detailed step-by-step instruction of the Nano-OTS protocol is available on protocols.io (https://www.protocols.io/view/nano-ots-bjmukk6w) [53].

### Alignment of reads and detection of off-target gRNA binding sites

The reads from SMRT-OTS and Nano-OTS were aligned to GRCh38 using minimap2 [54], after which gRNA binding sites were predicted using v1.9 of the Insider software [55]. For each predicted gRNA binding site, the corresponding sequence from GRCh38 was extracted in a ± 40 bp window surrounding the Cas9 cleavage site. All sequences containing gaps (N’s) were filtered out since we were only interested in detection of gRNA binding event in high-quality regions of the human genome. For the remaining sequences, we performed global alignment against all gRNA sequences using v6.6.0 of EMBOSS-Needle with default settings [56]. Only sequences with containing an alignment score of > 55 to a certain gRNA were considered positive binding sites.

### De novo assembly of HEK293 HiFi SMRT sequencing data

Data from two HiFi Sequel II SMRTcells were assembled with Peregrine build 0.1.6.0, using a docker image on an AWS r5d.12xlarge instance. The command options are available in Additional file 1: Supplementary Information.

### Molecular cloning and plasmid preparation

One micromolar of oligonucleotides for *NEK1* and *MMP14* with flanking *BpiI* restriction sites was ligated to dsDNA sgRNA’s by incubation with T4 Polynucleotide Kinase (EK0031) for 37 °C 30 min, 14x (95–25 °C 1 min) according to the manufacturer’s protocol (Thermo Scientific). The pSpCas9(BB)-2A-GFP vector (PX458, Addgene) was digested with FastDigest *BpiI* (FD1014, Thermo Scientific). Cloning of sgRNAs was performed using Rapid DNA Ligation Kit (K1422) according to the manufacturer’s protocol (Thermo Scientific). Vectors were purified using EndoFree Plasmid Maxi kit (Qiagen) and AMPure PB magnetic beads (Pacific Biosciences). The sgRNA sequence was amplified using *Taq* Polymerase chain reaction (PCR) on 50 ng vector DNA: 95 °C 5 min, 20x (95 °C 20 s, 65–55 °C 30 s, 72 °C 1 min) and 25x (95 °C 20 s, 55 °C 30 s, 72 °C 1 min) and confirmed using Sanger sequencing on a 3130XL ABI Genetic Analyzer using ABI Prism Big Dye Primer v3.0 Cycle Sequencing Ready Reaction with forward (5′ GAG GGC CTA TTT CCC ATG ATT) and reversed (5′ CAC GCG CYA AAA ACG GAC TA) primers according to the manufacturer’s protocol (Applied Biosystems, Waltham, MA).

### Gene editing of human fibroblast cells using CRISPR-Cas9

A total of 10.2 × 10^6^ human primary dermal fibroblasts (ATCC, PCS-201-012, passage 9) were trypsinized using Trypsin-EDTA 0.05% phenol red (Thermo Fisher Scientific) and resuspended with buffer R to a concentration of 6 × 10^6^ cells/ml. Triplicates of 6 × 10^5^ cells were electroporated with 3 μg of plasmid DNA using Neon Transfection System 100-μl tip (Invitrogen) by 1650 V, 10 ms width and three pulses. Transfection efficiency was estimated 48 h post-transfection using Invitrogen EVOS FL 6 fluorescence microscopy (Thermo Fisher Scientific). Images were magnified × 10 with optimized contrast and brightness to detect GFP and allow semiquantitative analysis. DNA was extracted from transfected and control cells at passage nine and the reference genome at passage twelve using Nucleospin Tissue kit (Machery Nagel).

### Multiplexed long-range PCR for re-sequencing of edited cells

PCR primers were designed for on- and off-target sites for the *MMP14* and *NEK1* gRNAs. The amplicons were designed to be 4.1 to 8.6 kb (Additional file 1: Tables S9-S10). Multiplexed long-range PCRs were performed using the PrimeStar GLX Polymerase (Takara Bio) according to the manufacturer’s instructions. PCRs were performed using all *MMP14* and *NEK1* primers on wildtype fibroblast DNA as a control, and on *NEK1* and *MMP14* edited fibroblasts. The PCR products were sequenced on PacBio’s Sequel system, using the Template Preparation Kit 1.0 for SMRTbell construction and 3.0 sequencing and binding chemistry for sequencing using a 10-h movie time.

### Computational analyses of predicted on- and off targets in edited cells

CCS reads were created for the SMRT long-amplicon data, after which alignment was performed to GRCh38 using minimap2 [54]. Next, the number of insertion and deletion events was detected for each on-target and off-target site, and the number of insertions and deletions was calculated in a ± 2 kb window surrounding the Cas9 cleavage site using the mpileup command in SAMtools [57]. This extraction of indel events was performed both for the CRISPR-Cas9 edited fibroblast cells and for the unedited fibroblast cells. Finally, the percentages of inserted/deleted bases in the unedited cells were subtracted from the percentage of inserted/deleted bases in the CRISPR-Cas9 edited cells. The resulting fraction corresponds to the values shown in Fig. 6b, c.

## Supplementary information

**Additional file 1: Supplementary Material**. **Supplementary Tables**, **Figures and Information**.

**Additional file 2: Supplementary Data 1**. SMRT-OTS results for *ATXN10*, *MMP14* and *NEK1*.

**Additional file 3: Supplementary Data 2**. Nano-OTS results for *ATXN10*, *MMP14* and *NEK1*.

**Additional file 4: Supplementary Data 3**. Overlapping results between SMRT-OTS and Nano-OTS for *ATXN10*, *MMP14* and *NEK1*.

**Additional file 5: Supplementary Data 4**. Downsampled Nano-OTS results for *ATXN10*, *MMP14* and *NEK1*.

**Additional file 6: Supplementary Data 5**. CHOPCHOP Cas9 cleavage site predictions for *ATXN10*, *MMP14* and *NEK1*.

**Additional file 7: Supplementary Data 6**. Results for SMRT-OTS and Nano-OTS for *EMX1*, *FANCF*, *RNF2* and *VEGFA*.

**Additional file 8: Supplementary Data 7**. Nano-OTS results for single-plex run of *EMX1*, *FANCF*, *RNF2* and *VEGFA*.

**Additional file 9: Supplementary Data 8**. Nano-OTS results for *CRYAA*, *HSPA1A*, *IKBKG*, *OPN1LW*, *OTOA*, and *STRC.*

**Additional file 10: Supplementary Data 9**. SMRT-OTS results for *ATXN10*, *MMP14* and *NEK1* when using de novo assembled HEK293 genome for alignment.

**Additional file 11: Supplementary Data 10**. Results of PBSV structural variant calling for HEK293 HiFi data.

**Additional file 12: Supplementary Data 11**. Large insertions detected at the *MMP14* and *NEK1* on-target sites in edited fibroblasts.

**Additional file 13.** Review history.

## Data Availability

The datasets generated and analyzed during the current study are available in the Sequence Read Archive (NCBI) repository under accession PRJNA612419 [58]. The SMRT-OTS [52] and Nano-OTS [53] protocols are available from https://www.protocols.io. The Insider software is released under the GNU General Public License version 3 or later and is available from GitHub: https://github.com/UppsalaGenomeCenter/InSiDeR [59]. The source code used in this study is available from Zenodo (10.5281/zenodo.4159442) [55].

## References

[1] Cho SW, Kim S, Kim JM, Kim JS (2013). Targeted genome engineering in human cells with the Cas9 RNA-guided endonuclease. Nat Biotechnol.

[2] Cong L, Ran FA, Cox D, Lin S, Barretto R, Habib N, Hsu PD, Wu X, Jiang W, Marraffini LA, Zhang F (2013). Multiplex genome engineering using CRISPR/Cas systems. Science.

[3] Jinek M, East A, Cheng A, Lin S, Ma E, Doudna J (2013). RNA-programmed genome editing in human cells. Elife.

[4] Mali P, Yang L, Esvelt KM, Aach J, Guell M, DiCarlo JE, Norville JE, Church GM (2013). RNA-guided human genome engineering via Cas9. Science.

[5] Hsu PD, Lander ES, Zhang F (2014). Development and applications of CRISPR-Cas9 for genome engineering. Cell.

[6] Fellmann C, Gowen BG, Lin PC, Doudna JA, Corn JE (2017). Cornerstones of CRISPR-Cas in drug discovery and therapy. Nat Rev Drug Discov.

[7] Ormond KE, Mortlock DP, Scholes DT, Bombard Y, Brody LC, Faucett WA, Garrison NA, Hercher L, Isasi R, Middleton A (2017). Human Germline genome editing. Am J Hum Genet.

[8] Brokowski C, Adli M (2019). CRISPR ethics: moral considerations for applications of a powerful tool. J Mol Biol.

[9] Fu Y, Foden JA, Khayter C, Maeder ML, Reyon D, Joung JK, Sander JD (2013). High-frequency off-target mutagenesis induced by CRISPR-Cas nucleases in human cells. Nat Biotechnol.

[10] Hsu PD, Scott DA, Weinstein JA, Ran FA, Konermann S, Agarwala V, Li Y, Fine EJ, Wu X, Shalem O (2013). DNA targeting specificity of RNA-guided Cas9 nucleases. Nat Biotechnol.

[11] Mali P, Aach J, Stranges PB, Esvelt KM, Moosburner M, Kosuri S, Yang L, Church GM (2013). CAS9 transcriptional activators for target specificity screening and paired nickases for cooperative genome engineering. Nat Biotechnol.

[12] Haeussler M. CRISPR off-targets: a question of context. Cell Biol Toxicol. 2019;36:5–9. 10.1007/s10565-019-09497-1 PMC7056574 31734746

[13] Strohkendl I, Saifuddin FA, Rybarski JR, Finkelstein IJ, Russell R (2018). Kinetic basis for DNA target specificity of CRISPR-Cas12a. Mol Cell.

[14] Anzalone AV, Randolph PB, Davis JR, Sousa AA, Koblan LW, Levy JM, Chen PJ, Wilson C, Newby GA, Raguram A, Liu DR (2019). Search-and-replace genome editing without double-strand breaks or donor DNA. Nature.

[15] Jiang F, Doudna JA (2017). CRISPR-Cas9 structures and mechanisms. Annu Rev Biophys.

[16] Kosicki M, Tomberg K, Bradley A (2018). Repair of double-strand breaks induced by CRISPR-Cas9 leads to large deletions and complex rearrangements. Nat Biotechnol.

[17] Aryal NK, Wasylishen AR, Lozano G (2018). CRISPR/Cas9 can mediate high-efficiency off-target mutations in mice in vivo. Cell Death Dis.

[18] Iyer V, Boroviak K, Thomas M, Doe B, Riva L, Ryder E, Adams DJ (2018). No unexpected CRISPR-Cas9 off-target activity revealed by trio sequencing of gene-edited mice. PLoS Genet.

[19] Luo X, He Y, Zhang C, He X, Yan L, Li M, Hu T, Hu Y, Jiang J, Meng X (2019). Trio deep-sequencing does not reveal unexpected off-target and on-target mutations in Cas9-edited rhesus monkeys. Nat Commun.

[20] Bae S, Park J, Kim JS (2014). Cas-OFFinder: a fast and versatile algorithm that searches for potential off-target sites of Cas9 RNA-guided endonucleases. Bioinformatics.

[21] Haeussler M, Schonig K, Eckert H, Eschstruth A, Mianne J, Renaud JB, Schneider-Maunoury S, Shkumatava A, Teboul L, Kent J (2016). Evaluation of off-target and on-target scoring algorithms and integration into the guide RNA selection tool CRISPOR. Genome Biol.

[22] Labun K, Montague TG, Krause M, Torres Cleuren YN, Tjeldnes H, Valen E (2019). CHOPCHOP v3: expanding the CRISPR web toolbox beyond genome editing. Nucleic Acids Res.

[23] Cameron P, Fuller CK, Donohoue PD, Jones BN, Thompson MS, Carter MM, Gradia S, Vidal B, Garner E, Slorach EM (2017). Mapping the genomic landscape of CRISPR-Cas9 cleavage. Nat Methods.

[24] Kim D, Bae S, Park J, Kim E, Kim S, Yu HR, Hwang J, Kim JI, Kim JS (2015). Digenome-seq: genome-wide profiling of CRISPR-Cas9 off-target effects in human cells. Nat Methods.

[25] Kim D, Kim S, Kim S, Park J, Kim JS (2016). Genome-wide target specificities of CRISPR-Cas9 nucleases revealed by multiplex Digenome-seq. Genome Res.

[26] Lazzarotto CR, Malinin NL, Li Y, Zhang R, Yang Y, Lee G, Cowley E, He Y, Lan X, Jividen K, et al. CHANGE-seq reveals genetic and epigenetic effects on CRISPR-Cas9 genome-wide activity. Nat Biotechnol. 2020;38:1317–27. 10.1038/s41587-020-0555-7 PMC7652380 32541958

[27] Lazzarotto CR, Nguyen NT, Tang X, Malagon-Lopez J, Guo JA, Aryee MJ, Joung JK, Tsai SQ (2018). Defining CRISPR-Cas9 genome-wide nuclease activities with CIRCLE-seq. Nat Protoc.

[28] Tsai SQ, Nguyen NT, Malagon-Lopez J, Topkar VV, Aryee MJ, Joung JK (2017). CIRCLE-seq: a highly sensitive in vitro screen for genome-wide CRISPR-Cas9 nuclease off-targets. Nat Methods.

[29] Ameur A, Kloosterman WP, Hestand MS (2019). Single-molecule sequencing: towards clinical applications. Trends Biotechnol.

[30] Audano PA, Sulovari A, Graves-Lindsay TA, Cantsilieris S, Sorensen M, Welch AE, Dougherty ML, Nelson BJ, Shah A, Dutcher SK (2019). Characterizing the major structural variant alleles of the human genome. Cell.

[31] Chaisson MJP, Sanders AD, Zhao X, Malhotra A, Porubsky D, Rausch T, Gardner EJ, Rodriguez OL, Guo L, Collins RL (2019). Multi-platform discovery of haplotype-resolved structural variation in human genomes. Nat Commun.

[32] Ebbert MTW, Jensen TD, Jansen-West K, Sens JP, Reddy JS, Ridge PG, Kauwe JSK, Belzil V, Pregent L, Carrasquillo MM (2019). Systematic analysis of dark and camouflaged genes reveals disease-relevant genes hiding in plain sight. Genome Biol.

[33] Wenger AM, Peluso P, Rowell WJ, Chang PC, Hall RJ, Concepcion GT, Ebler J, Fungtammasan A, Kolesnikov A, Olson ND (2019). Accurate circular consensus long-read sequencing improves variant detection and assembly of a human genome. Nat Biotechnol.

[34] Tsai Y-C, Greenberg D, Powell J, Höijer I, Ameur A, Strahl M, Ellis E, Jonasson I, Mouro Pinto R, Wheeler VC, et al: Amplification-free, CRISPR-Cas9 targeted enrichment and SMRT sequencing of repeat-expansion disease causative genomic regions. bioRxiv. 2017:203919.

[35] Hoijer I, Tsai YC, Clark TA, Kotturi P, Dahl N, Stattin EL, Bondeson ML, Feuk L, Gyllensten U, Ameur A (2018). Detailed analysis of HTT repeat elements in human blood using targeted amplification-free long-read sequencing. Hum Mutat.

[36] Gilpatrick T, Lee I, Graham JE, Raimondeau E, Bowen R, Heron A, Downs B, Sukumar S, Sedlazeck FJ, Timp W (2020). Targeted nanopore sequencing with Cas9-guided adapter ligation. Nat Biotechnol.

[37] Stangl C, de Blank S, Renkens I, Westera L, Verbeek T, Valle-Inclan JE, Gonzalez RC, Henssen AG, van Roosmalen MJ, Stam RW (2020). Partner independent fusion gene detection by multiplexed CRISPR-Cas9 enrichment and long read nanopore sequencing. Nat Commun.

[38] Robinson JT, Thorvaldsdottir H, Winckler W, Guttman M, Lander ES, Getz G, Mesirov JP (2011). Integrative genomics viewer. Nat Biotechnol.

[39] Ameur A, Dahlberg J, Olason P, Vezzi F, Karlsson R, Martin M, Viklund J, Kahari AK, Lundin P, Che H (2017). SweGen: a whole-genome data resource of genetic variability in a cross-section of the Swedish population. Eur J Hum Genet.

[40] Shi L, Guo Y, Dong C, Huddleston J, Yang H, Han X, Fu A, Li Q, Li N, Gong S (2016). Long-read sequencing and de novo assembly of a Chinese genome. Nat Commun.

[41] Ameur A, Che H, Martin M, Bunikis I, Dahlberg J, Hoijer I, Haggqvist S, Vezzi F, Nordlund J, Olason P, et al. De novo assembly of two Swedish genomes reveals missing segments from the human GRCh38 reference and improves variant calling of population-scale sequencing data. Genes (Basel). 2018;9:486. 10.3390/genes9100486 PMC6210158 30304863

[42] Chin C-S, Khalak A: Human genome assembly in 100 minutes. bioRxiv. 2019:705616.

[43] Heller D, Vingron M (2019). SVIM: structural variant identification using mapped long reads. Bioinformatics.

[44] Miga KH, Koren S, Rhie A, Vollger MR, Gershman A, Bzikadze A, Brooks S, Howe E, Porubsky D, Logsdon GA (2020). Telomere-to-telomere assembly of a complete human X chromosome. Nature..

[45] Tsai SQ, Zheng Z, Nguyen NT, Liebers M, Topkar VV, Thapar V, Wyvekens N, Khayter C, Iafrate AJ, Le LP (2015). GUIDE-seq enables genome-wide profiling of off-target cleavage by CRISPR-Cas nucleases. Nat Biotechnol.

[46] Wienert B, Wyman SK, Richardson CD, Yeh CD, Akcakaya P, Porritt MJ, Morlock M, Vu JT, Kazane KR, Watry HL (2019). Unbiased detection of CRISPR off-targets in vivo using DISCOVER-Seq. Science.

[47] Scott DA, Zhang F (2017). Implications of human genetic variation in CRISPR-based therapeutic genome editing. Nat Med.

[48] Chen CL, Rodiger J, Chung V, Viswanatha R, Mohr SE, Hu Y, Perrimon N (2020). SNP-CRISPR: a web tool for SNP-specific genome editing. G3 (Bethesda).

[49] Wu Y, Zeng J, Roscoe BP, Liu P, Yao Q, Lazzarotto CR, Clement K, Cole MA, Luk K, Baricordi C (2019). Highly efficient therapeutic gene editing of human hematopoietic stem cells. Nat Med.

[50] Kim MY, Yu KR, Kenderian SS, Ruella M, Chen S, Shin TH, Aljanahi AA, Schreeder D, Klichinsky M, Shestova O (2018). Genetic inactivation of CD33 in hematopoietic stem cells to enable CAR T cell immunotherapy for acute myeloid leukemia. Cell.

[51] Akcakaya P, Bobbin ML, Guo JA, Malagon-Lopez J, Clement K, Garcia SP, Fellows MD, Porritt MJ, Firth MA, Carreras A (2018). In vivo CRISPR editing with no detectable genome-wide off-target mutations. Nature.

[52] Höijer I, Johansson J, Gudmundsson S, Chin C-S, Bunikis I, Häggqvist S, Emmanouilidou A, Wilbe M, Md H, Bondeson M-L (2020). SMRT-OTS protocol description.

[53] Höijer I, Johansson J, Gudmundsson S, Chin C-S, Bunikis I, Häggqvist S, Emmanouilidou A, Wilbe M, Md H, Bondeson M-L (2020). Nano-OTS protocol description.

[54] Li H (2018). Minimap2: pairwise alignment for nucleotide sequences. Bioinformatics.

[55] Bunikis I, Ameur A. Insider - integration & celavage site detection. Zenodo. 2020. 10.5281/zenodo.4159442. Accessed 1 November 2020.

[56] Madeira F, Park Ym, Lee J, Buso N, Gur T, Madhusoodanan N, Basutkar P, Tivey ARN, Potter SC, Finn RD, Lopez R. The EMBL-EBI search and sequence analysis tools APIs in 2019. Nucleic Acids Res. 2019;47(W1):W636–41. 10.1093/nar/gkz268 PMC6602479 30976793

[57] Li H, Handsaker B, Wysoker A, Fennell T, Ruan J, Homer N, Marth G, Abecasis G, Durbin R, Genome Project Data Processing S (2009). The sequence alignment/map format and SAMtools. Bioinformatics.

[58] Höijer I, Johansson J, Gudmundsson S, Chin C-S, Bunikis I, Häggqvist S, Emmanouilidou A, Wilbe M, Hoed Md, Bondeson M-L, et al: SMRT-OTS and Nano-OTS sequence data. Datasets. Sequence Read Archive https://www.ncbi.nlm.nih.gov/bioproject/PRJNA612419/. 2020. Accessed 1 November 2020.

[59] Bunikis I, Ameur A: Insider - integration & cleavage site detection. Github. 2020. https://github.com/UppsalaGenomeCenter/InSiDeR. Accessed 1 November 2020.

