# Supplementary material for: Amplification-free long-read sequencing reveals unforeseen CRISPR-Cas9 off-target activity
Source: Genome Biol. 2020 Dec 1;21:290. doi: 10.1186/s13059-020-02206-w (PMC7706270; doi:10.1186/s13059-020-02206-w)
Supplement: Supplementary file 1 — Additional file 1: Supplementary Material. Supplementary Tables, Figures and Information. [file 13059_2020_2206_MOESM1_ESM.pdf]

Supplementary Material

Amplification-free long read sequencing reveals unforeseen CRISPR-Cas9 off-target activity in vitro

Ida Höijer, Josefin Johansson, Sanna Gudmundsson, Chen-Shan Chin, Ignas Bunikis, Susana Häggqvist, Anastasia Emmanouilidou, Maria Wilbe, Marcel den Hoed, Marie-Louise Bondeson, Lars Feuk, Ulf Gyllensten, Adam Ameur

Table of contents

Supplementary Figures 2
Supplementary Tables...5
Supplementary Information 11
De novo assembly of PacBio HiFi data 11
SV detection in long amplicon re-sequencing data...11

Supplementary Figures

Mapped CCS read statistics

Value	Analysis Metric
98.54%	Mapped CCS Read Mean Concordance
3,702,538	Number of Alignments
3,562,153	Number of CCS Reads (mapped)
55,717,061,882	Number of CCS Bases (mapped)
15,048	CCS Read Length Mean (mapped)
15,310	CCS Read Length N50 (mapped)
20,480	CCS Read Length 95% (mapped)
36,684	CCS Read Length Max (mapped)

Mapped CCS read length

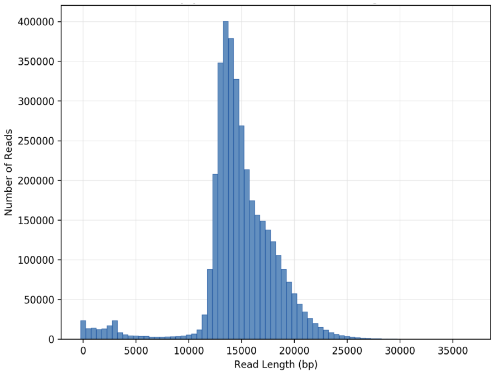

Figure S1. Throughput and alignment statistics for HiFi data for the HEK293 cell line used in this study. The HEK293 DNA was sequenced on two Sequel II SMRT cells, after which CCS analysis was performed. The panels show the results after alignment to the human GRCh38 reference.

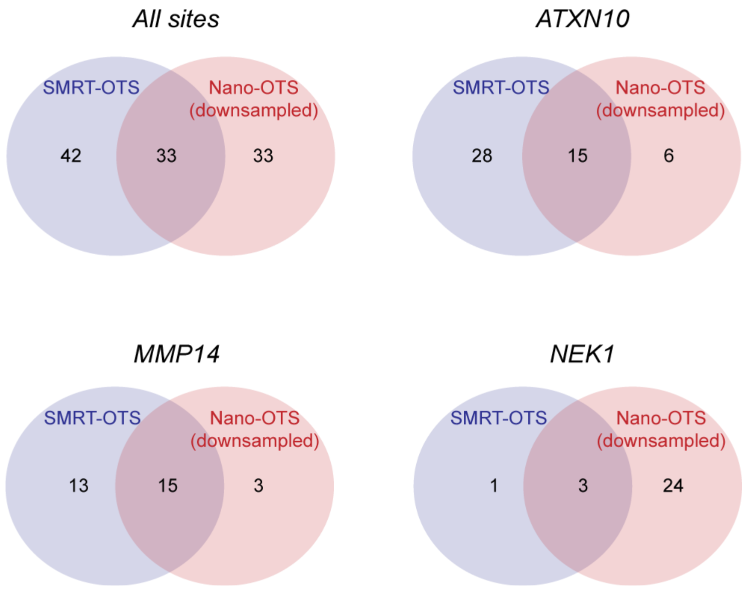

Figure S2. Venn diagrams showing the overlap between Cas9 cleavage sites predicted for SMRT-OTS and downsampled Nano-OTS data. The top left panel shows results for the three gRNAs (*ATXN10*, *MMP14* and *NEK1*) combined, and the three other panels show results for the individual gRNAs. In this comparison, the Nano-OTS data has been downsampled to the same number of reads as the SMRT-OTS data.

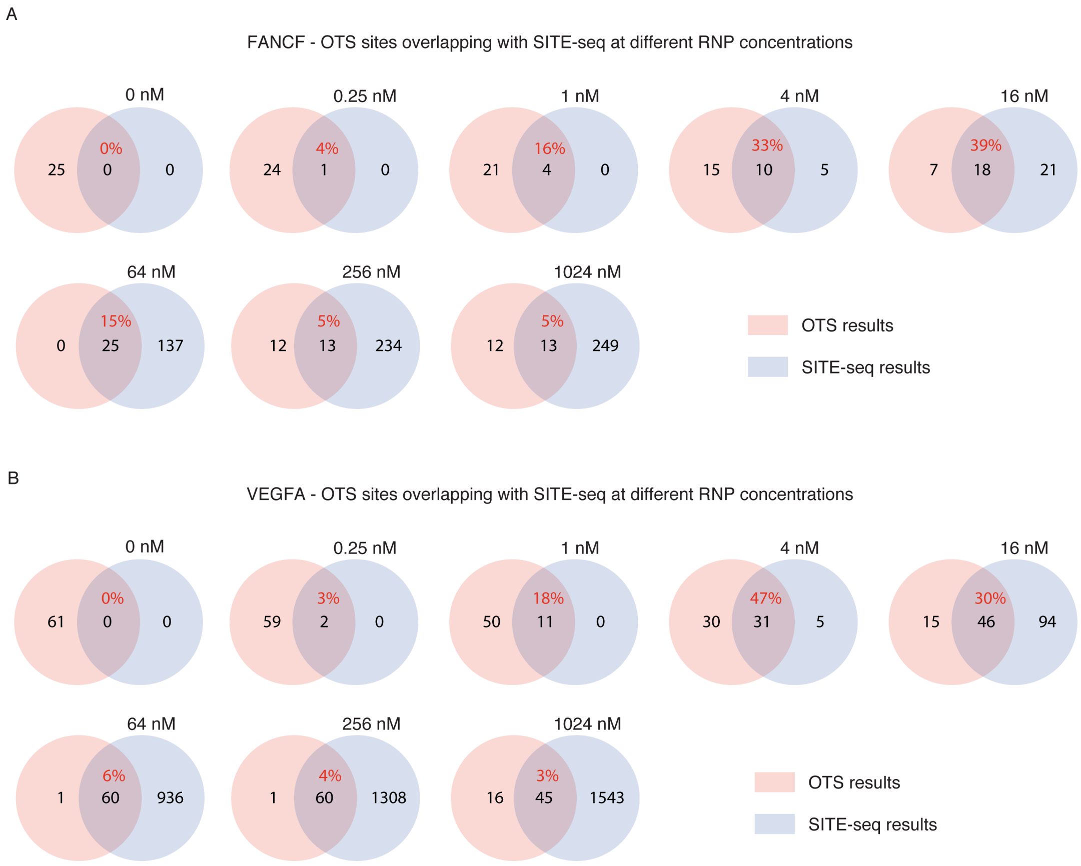

Figure S3. Comparison of OTS to SITE-seq. A) The Venn diagrams show the overlap between the 25 *FANCF* OTS sites and SITE-seq results using the same gRNA at different RNP concentrations. B) Overlap between the 61 *VEGFA* OTS sites and SITE-seq results using the same gRNA at different RNP concentrations.

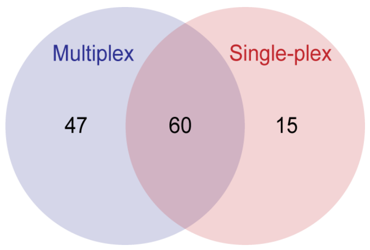

Figure S4. Comparison of Nano-OTS Cas9 cleavage sites detected for the gRNAs EMX1, FANCF, RNF2 and VEGFA in a multiplex run (left) and in a single-plex run (right). 60 Cas9 cleavage sites were detected in both runs.

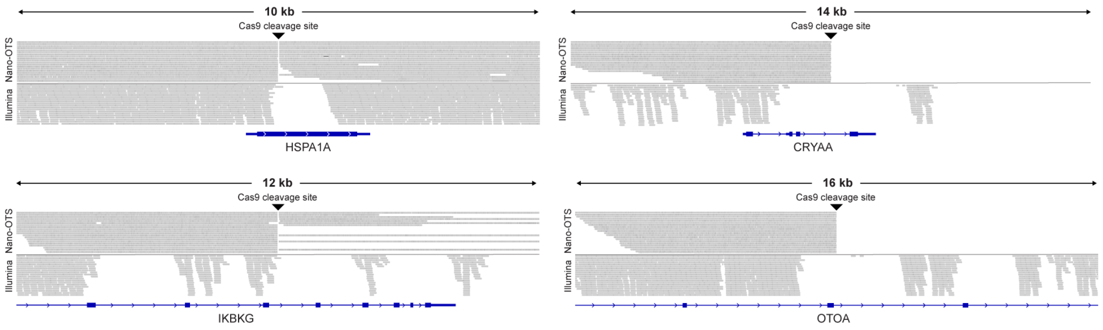

Figure S5. Four examples of "dark" genomic regions, *HSPA1A*, *CRYAA*, *IKBKG* and *OTOA* where Nano-OTS successfully identified an on-target site while short-read data failed to uniquely align. In each panel, the Nano-OTS reads are displayed at the top and at the bottom is data from one individual from the SweGen dataset.

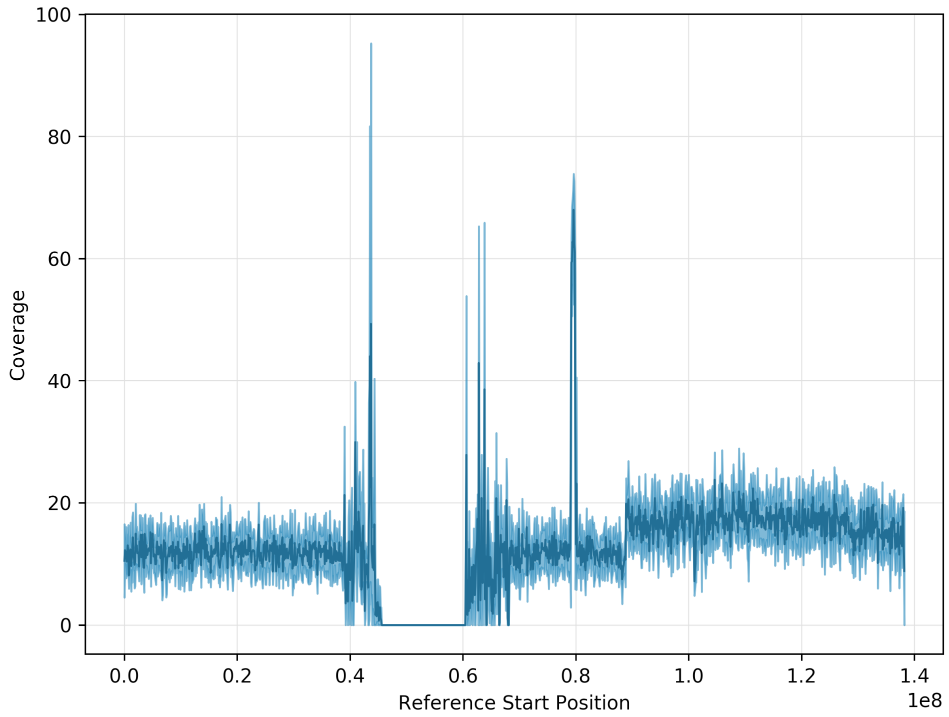

Figure S6. Coverage profile of HEK293 HiFi data on chromosome 9. The SNP rs7861875 (chr9:109,570,956) is located near coordinate 1.1 in a part of the chromosome that has elevated coverage as compared to the rest of the chromosome.

Supplementary Tables

Table S1. Sequences and genomic coordinates for the gRNAs used in this study.

Target	Sequence	Genomic coordinates (GRCh38)
ATXN10	AUACAAAGGAUCAGAAUCCC	chr22:45794847-45794866
MMP14	GCGGUGCCGAGCGUGAGCAG	chr14:22836860-22836879
NEK1	AUAGUAAUGGAUUACUGUGA	chr4:169599161-169599180
EMX1	GAGUCCGAGCAGAAGAAGAA	chr2:72933853-72933872
FANCF	GGAAUCCCUUCUGCAGCACC	chr11:22625789-22625808
RNF2	GUCAUCUUAGUCAUUACCUG	chr1:185087638-185087657
VEGFA[a]	GGGUGGGGGGAGUUUGCUCC	chr6:43769557-43769576
CRYAA	GGCUACGUGGGGGCUUUACC	chr21:43171532-43171551
HSPA1A	UGAUCACCGCGUUGGUCACC	chr6:31816170-31816189
IKBKG	ACACGCAGUGAAACGUGGUC	chrX:154560786-154560805
OPN1LW	UACACUAUCGGGGUAGUGCC	chrX:154151838-154151857
OTOA	UAUGGGAAAACUGCUCGAGG	chr16:21740996-21741015
STRC	ACGGACGAUUGGGGAAUACC	chr15:43615531-43615550

[a] The VEGFA gRNA is also known by the names "VEGFA site 1" and "VEGFA1" from previous studies

Table S2. OTS sites found both by SMRT-OTS and Nano-OTS, but not detected by CHOPCHOP

chr	position	gRNA	DNA binding sequence	mm	indels	SMRT	Nano
chr1	199581904	atxn10	TAATAAAGGATCAGAATACCAGG	4	0	45	558
chr7	132754767	atxn10	ATAC-TAGGATCAAAATCCCAGG	2	1	35	276
chr2	13854278	atxn10	CCAGGGATTCTGATCCATGTCCAT	3	1	17	25
chr7	146700912	atxn10	CAACAAAGGATAAGAATCCCAAG	4	0	14	30
chr2	30757626	atxn10	ATGCTCAGGATCAGAATCCCATG	4	0	12	31
chr5	60703887	atxn10	CATGGGATTCTGATCCTCT-TCT	3	1	10	41
chr10	104676047	atxn10	CCAGGGA-TCTGATGCTTTGTGA	3	1	9	38
chr2	14708587	atxn10	CCAGGGATTCTGTTCCTGAGTGT	4	0	8	19
chr8	23569322	atxn10	ACCTACTGGATCAGAATCCCTGG	5	0	8	17
chr20	17814888	atxn10	TTAGCATGGATCAGAATCCCGGG	4	0	7	7
chr2	216830092	atxn10	ACCTACAGAATCAGAATCCCTGG	5	0	7	11
chr15	73766400	atxn10	ATCCAAAGGATACAGAATCCCAGG	1	1	6	30
chr8	135042874	atxn10	CCAGGGA-TCTGATCCTCTGCAG	3	1	5	8
chr5	139887571	mmp14	GCAGTGCCAAGCATGAGTAGTGG	4	0	75	115
chrX	75180043	mmp14	GAGGTGCCAAGAGTGAGCAAGGG	4	0	50	75
chr2	1759836	mmp14	GAGGTGCTGAGAGTGAGCAAGGG	4	0	49	201
chr3	106643626	mmp14	CCCTTGCTCACTCTTGGCACCTC	4	0	39	66
chr1	26500687	mmp14	CCCCTGCTCAGGCTCGGCTCAAC	4	0	26	204
chr7	50393714	mmp14	CCTGTGCTGAGCGTAAGCAGTGG	4	0	17	13
chr13	27212020	mmp14	CCCTTGCTCACTCTTGGCACCTC	4	0	16	26
chr10	5943738	mmp14	CCCCTGCTCATGTTCGGTGCCTC	5	0	11	85
chr13	54676451	mmp14	CCCTTGCTCACTCTCGGCGCCGA	4	0	9	22
chr2	72638487	mmp14	AAGGTACCGAGAGTGAACAGAGG	5	0	9	43
chr20	1178970	nek1	CCATCACAGTAATCCATATGACT	5	0	16	346
chr8	135438302	nek1	CCTTCACAGTAATCCAATGAAGTAT	2	2	5	25

Table S3. Four OTS-sites with >1% genome editing both from the Digenome-seq and CIRCLE-seq study

chr	pos	sequence	Digenome-seq (% edited)[a]	CIRCLE-seq (% edited)[b]	target
chr8	127789014	GAGTCCtAGCAGgAGAAGAAGAG	6.67 (HeLa)	1.30 (K562)	EMX1 off-target
chr2	218980351	GAGgCCGAGCAGAAGAAagACGG	6.38 (HeLa)	2.94 (K562)	EMX1 off-target
chr15	65345199	GGaTGGaGGGAGTTTGCTCCTGG	25.28 (HeLa)	9.86 (U2OS)	VEGFA off-target
chr12	1878911	cGGgGGaGGGAGTTTGCTCCTGG	11.73 (HeLa)	2.60 (U2OS)	VEGFA off-target

[a] HeLa cells were used for genome editing in the Digenome-seq study

[b] K562 and U2OS cells were used for genome editing in the CIRCLE-seq study

Table S4. Identified on- and off-targets for a Nano-OTS with six gRNAs designed in six "dark" genic regions: *CRYAA*, *HSPA1A*, *IKBKG*, *OPN1LW*, *OTOA*, and *STRC*.

chromosome	start	end	gRNA	on/off-target	in dark region[a]
chr21	6563217	6563217	CRYAA	off-target	yes
chr21	43171533	43171533	CRYAA	on-target	yes
chrX	79689123	79689123	CRYAA	off-target	no
chr1	25413070	25413070	CRYAA	off-target	no
chr2	60220583	60220583	CRYAA	off-target	no
chr10	128399880	128399880	CRYAA	off-target	no
chrX	83128564	83128564	CRYAA	off-target	no
chr1	49353600	49353600	CRYAA	off-target	no
chr10	121130357	121130357	CRYAA	off-target	no
chrX	107482006	107482006	CRYAA	off-target	no
chr1	223039522	223039522	CRYAA	off-target	no
chr3	127921470	127921470	CRYAA	off-target	no
chr6	31816171	31816171	HSPA1A	on-target	yes
chr6	31828353	31828366	HSPA1A	off-target	yes
chr21	46567455	46567455	HSPA1A	off-target	no
chr17	30212855	30212855	HSPA1A	off-target	no
chrX	154643649	154643649	IKBKG	off-target	yes
chrX	154560788	154560788	IKBKG	on-target	yes
chrX	154188955	154188955	OPN1LW	off-target	no
chrX	154226093	154226093	OPN1LW	off-target	yes
chrX	154263897	154263897	OPN1LW	off-target	yes
chrX	154151841	154151841	OPN1LW	on-target	yes
chr7	17319262	17319262	OPN1LW	off-target	no
chr16	21740996	21740996	OTOA	on-target	yes
chr16	22557118	22557118	OTOA	off-target	yes
chr2	137384792	137384799	OTOA	off-target	no
chr3	177404425	177404425	OTOA	off-target	no
chr15	43615532	43615532	STRC	on-target	yes
chr15	43714994	43714994	STRC	off-target	yes
chr10	128550395	128550395	STRC	off-target	no

[a] The value in this column is "yes" if the target site overlaps with dark regions from the study by Ebbery et al, 2019: Ebbert, M.T.W., Jensen, T.D., Jansen-West, K. *et al.* Systematic analysis of dark and camouflaged genes reveals disease-relevant genes hiding in plain sight. *Genome Biol* **20,** 97 (2019). https://doi.org/10.1186/s13059-019-1707-2. Overlap was calculated using BEDTools using default settings.

Table S5. Results of *de novo* assembly of HEK293 HiFi data

	**HEK293 *de novo* assembly statistics**
Total contigs	2,110
Total bases	2,896,467,641
Max contig length	47,094,793
N50	11,221,406
N90	1,874,419
N95	724,820

Table S6. Unique reads supporting large insertion/deletion events (>50bp) at *MMP14* and *NEK1* on-target sites in CRISPR-Cas9 edited fibroblast cells

**On-target**	**Large insertion/deletion variant**	**SMRT cell and read ID**
MMP14	DEL 623	m54259_200128_194006/72679552/ccs
MMP14	DEL 287	m54259_200128_194006/31719500/ccs
MMP14	INS 293 CGTTGGGCAGGTTCTTATCGAAGTT...GGGATGC GGAAGGTCAGGATCTTCT	m54259_200128_194006/57737325/ccs
MMP14	DEL 87	m54259_200128_194006/61669573/ccs
MMP14	DEL 103	m54259_200128_194006/25428036/ccs
MMP14	DEL 261	m54259_200128_194006/25952997/ccs
MMP14	DEL 61	m54259_200128_194006/28509175/ccs
MMP14	INS 382 AGGGGATGCGGAAGGTCAGGATCTT...TTTGTAC TTCTCAGGCAGCTGCTGC	m54259_200128_194006/19137446/ccs
MMP14	INS 75 GCTGTGCTTTCTTTAAAGTTTCAAA...GTATCTGA AGATGAATGTTTTGAGT	m54259_200128_194006/17826396/ccs
MMP14	DEL 122	m54259_200128_194006/15598537/ccs
MMP14	INS 591 AGCGGGGCGGGGCGGGGCGAGGGGC...GTGAT CACCGACGAGTACAAGGTGC	m54259_200128_194006/53936325/ccs
MMP14	INS 98 GAACTTCAGGGTCAGCTTGCCGTAG...ACGTCGC CGTCCAGCTCGACCAGGA	m54259_200128_194006/26345740/ccs
MMP14	INS 324 TGAGGGGATGCGGAAGGTCAGGATC...AAATCTC TTTGTACTTCTCAGGCAG	m54259_200128_194006/22086087/ccs
MMP14	DEL 371	m54259_200128_194006/25232004/ccs
MMP14	INS 247 AGATTATCAAAAAGGATCTTCACCT...GCAATGAT ACCGCGGCTTCCACGCT	m54259_200128_194006/37749032/ccs
MMP14	DEL 122	m54259_200128_194006/20775878/ccs
MMP14	INS 198 TGTCTCAACAGCGGTAAGATCCTTG...CAGTCACA GAAAAGCATCTTACGGA	m54259_200128_194006/10813796/ccs
NEK1	DEL 69	m54259_200129_161744/10814348/ccs
NEK1	DEL 65	m54259_200129_161744/56099349/ccs
NEK1	INS 262 TGGCCGACAAGCAGAAGAACGGCAT...GGCATG GACGAGCTGTACAAGGAAT	m54259_200129_161744/73597357/ccs
NEK1	INS 333 AGCCCTTGTCCACCACTTCCTCGAA...ATCTTTTC CAGGATGGGCTTGATGA	m54259_200129_161744/42468304/ccs

[a] 17 of 5233 reads (0.324%) contained a large insertion/deletion at the *MMP14* on-target in edited cells. 0 of 18538 reads contained a large insertion/deletion at the *MMP14* on-target in control DNA

[b] 4 of 10404 reads (0.038%) contained a large insertion/deletion at the *NEK1* on-target in edited cells. 0 of 12998 reads contained a large insertion/deletion at the *NEK1* on-target in control DNA

Table S7. Number of insertion/deletion events identified at on- and off-target sites for *MMP14* in CRISPR-Cas9 edited fibroblast cells and in unedited control cells

		Insertion/deletion events in edited *MMP14* fibroblast cells[a]	Insertion/deletion events in control (unedited) fibroblast cells[b]
On-target	chr14:22836862	302 indels in 5310 reads (5.69%)	76 indels in 8095 reads (0.94%)
Off-target	chr10:30769946	31 indels in 3484 reads (0.90%)	109 indels in 7214 reads (1.51%)
Off-target	chr11:115160826	259 indels in 4457 reads (5.81%)	452 indels in 7968 reads (5.67%)
Off-target	chr3:166423833	35 indels in 1950 reads (1.79%)	131 indels in 7995 reads (1.63%)
Off-target	chr4:67287221	43 indels in 8057 reads (0.53%)	99 indels in 8117 reads (1.22%)
Off-target	chr5:50292712	94 indels in 7206 reads (1.30%)	115 indels in 7088 reads (1.62%)
Off-target	chr7:116998522	33 indels in 4460 reads (0.73%)	127 indels in 8023 reads (1.58%)
Off-target	chr5:139887571	16 indels in 7265 reads (0.21%)	43 indels in 7741 reads (0.55%)
Off-target	chr5:168537900	1 indels in 1037 reads (0.10%)	23 indels in 1625 reads (1.42%)
Off-target	chr6:67722906	21 indels in 7617 reads (0.28%)	66 indels in 7053 reads (0.94%)
Off-target	chr2:1759836	4 indels in 1521 reads (0.26%)	6 indels in 1797 reads (0.33%)
Off-target	chr7: 50393714	21 indels in 8033 reads (0.26%)	143 indels in 8088 reads (1.77%)
Off-target	chr21:22597662	28 indels in 8133 reads (0.34%)	74 indels in 8119 reads (0.91%)
Off-target	chr19:19626751	29 indels in 6736 reads (0.43%)	78 indels in 5318 reads (1.47%)
Off-target	chrX:75180043	25 indels in 8398 reads (0.30%)	23 indels in 8193 reads (0.28%)
Off-target	chr3:106643626	35 indels in 7911 reads (0.44%)	36 indels in 7789 reads (0.46%)

[a,b] The events correspond to all SMRT re-sequencing reads having an insertion or deletion start coordinate within a +-20bp window surrounding the Cas9 cleavage site

Table S8. Number of insertion/deletion events identified at on- and off-target sites for *NEK1* in CRISPR-Cas9 edited fibroblast cells and in unedited control cells

		Insertion/deletion events in edited *NEK1* fibroblast cells[a]	Insertion/deletion events in control (unedited) fibroblast cells[b]
On-target	chr4:169599163	721 indels in 8008 reads (9.00%)	21 indels in 8056 reads (0.26%)
Off-target	chr20:1178970	15 indels in 7914 reads (0.19%)	21 indels in 7982 reads (0.26%)
Off-target	chr8:122196851	0 indels in 386 reads (0%)	1 indels in 632 reads (0.16%)
Off-target	chr8:135438302	16 indels in 5751 reads (0.28%)	24 indels in 7757 reads (0.31%)

[a,b] The events correspond to all SMRT re-sequencing reads having an insertion or deletion start coordinate within a +-20bp window surrounding the Cas9 cleavage site

Table S9. Primer information for *MMP14* on- and off-target sites

On/off-target	Genomic coordinates (GRCh38)	Forward primer	Reverse primer	Amplicon length (bp)
On-target	chr14:22836862	CAGGACAGGAACCAGTAGTAGA	CAGAACGACAGAAGGAAGATGG	5146
Off-target	chr10:30769946	GGCTTGGACACCGAGATTAG	GAAGGGATGAAGCAGGAAGAG	5290
Off-target	chr11:115160826	GCACCCTACCCTTGTCTTATTT	AAGGAGAGGGTGTTGGTTTATG	5332
Off-target	chr3:166423833	TGTCTGGTCCTGGGCTATTT	GGCTCTTGTTCCATTCCCTTAC	5468
Off-target	chr4:67287221	TTCGTGCCTGTCCACATTAG	GTTAGCAGCGGTGTATCTGTAT	5049
Off-target	chr5:50292712	CATCGGGACATGACAATGATCTA	AGCAAAGCCTGCAAGAAATATG	5287
Off-target	chr7:116998522	CTGTGAGGGAATGTGGGTTATC	CTCCTGCTTTCTTCCCTTCATC	5434
Off-target	chr5:139887571	CAGGGAGGCCAGATAAGAATTG	GCCATCATCCCAAGAGGTAAG	5008
Off-target	chr5:168537900	GACAAGGAACAGACAGAGGAAG	TTGGGAACAGAGAATCCAAGAG	4624
Off-target	chr6:67722906	CATCCATCGACACAAGCTACA	GAGGAAGCATAGCTGGAACTAC	5070
Off-target	chr2:1759836	CTTGGTCCAACACCACCTCA	CCTCAGCAAAGGCTGGGTAT	8682
Off-target	chr7: 50393714	CAGAAGACCTGTGCAAGATAGG	TAGTGGGAAGAAGGCTGAAATG	4924
Off-target	chr21:22597662	AAGCCCAGGTGTCAAGAATAG	TAGCCCTTCCCAAGAACTAAAC	5074
Off-target	chr19:19626751	CACAGGGCTTACCAAGGATAC	GATCCGGTCCTGCATAATCAA	5012
Off-target	chrX:75180043	GGTCGGATTTAGTGGTCCTTAC	CATTCCACGCTCACAGATAAGA	4128
Off-target	chr3:106643626	GCAGTGTGGTGGGCTAAATA	GCTCTGTGACTAGGTGCATATC	5308

Table S10. Primer information for *NEK1* on- and off-target sites

On/off-target	Genomic coordinates (GRCh38)	Forward primer	Reverse primer	Amplicon length (bp)
On-target	chr4:169599163	CTGTCCAAGGGAACGTACTTAG	CTCCCAACTGTGCCTGTATAAT	5237
Off-target	chr20:1178970	CCCATCCTGTGGCTTCTTAAT	GGACCTCTGCTGTCCTTATTG	4486
Off-target	chr8:122196851	GCCCTTGTAGCAGTGAGATAC	CTCTCTAGAAACCCGCAAAGAG	4642
Off-target	chr8:135438302	GGGTGTAGGGCAACTTGATTAG	GCAGCTCCAAGTGTGAAAGA	5200

Supplementary Information

De novo assembly of PacBio HiFi data

SMRTcell data was assembled with Peregrine build 0.1.6.0 docker image with the following command options on an AWS r5d.12xlarge instance:

docker run -it -v /wd:/wd --user $(id -u):$(id -g) cschin/peregrine:0.1.6.0 asm /wd/seqdata.lst 24 24 24 24 24 24 24 24 24 --with-consensus --shimmer-r 3 --best_n_ovlp 8 --output /wd/asm

The file /wd/seqdata.lst contains the file path to the input file:

/wd/data/m64077_191107_133924.Q20.fasta.gz
/wd/data/m64077_191108_195203.Q20.fasta.gz

SV detection in long amplicon re-sequencing data

The software SVIM (v 1.4.0) was used for detection of large structural variants in the SMRT amplicon CCS data, with following parameters:

```
svim alignment --partition_max_distance 1 --insertion_sequences -
-types DEL,INS,INV --heterozygous_threshold 0.000001 --read_names
--min_sv_size 50 [CCS bamfile] [hg38 reference file]
```
