# Supplementary material for: Amplification-free long-read sequencing reveals unforeseen CRISPR-Cas9 off-target activity
Source: Genome Biol. 2020 Dec 1;21:290. doi: 10.1186/s13059-020-02206-w (PMC7706270; doi:10.1186/s13059-020-02206-w)
Supplement: Supplementary file 13 — Additional file 13. Review history. [file 13059_2020_2206_MOESM13_ESM.docx]

Review History

**First round of review**

**Reviewer 1**

**Are you able to assess all statistics in the manuscript, including the appropriateness of statistical tests used?** There are no statistics in the manuscript.

**Comments to author:**

In "Amplification-free long read sequencing reveals unforeseen CRISPR-Cas9 off-target activity in vitro", Hoijer et al. described the development of SMRT-OTS and Nano-OTS, two orthogonal methods for identifying Cas9 genome-wide off-target activity in vitro, leveraging long-read sequencing platforms. The workflow for both methods is straightforward and use molecular biology strategies similar to CIRCLE-seq and SITE-seq for selective sequencing of Cas9 cleaved DNA molecules. However, the overall novelty of the methods and conclusions is unclear from the manuscript in its current form and, there a number of points that should be addressed to warrant publication in Genome Biology.

Major
1 - Although the strategies described appear straightforward, the authors did not perform any direct comparison with established methods by analyzing known benchmark sgRNAs targets such as VEGFA, RNF2 and EMX1, which makes it difficult to access the relative  performance of SMRT-OTS and Nano-OTS. For completeness, target sites as the ones mentioned above could be evaluated for a fair comparison with published in vitro methods, such as CIRCLE-seq, Digenome-seq and SITE-seq. Indeed, if those targets could be evaluated, it would also be relevant to compare with GUIDE-seq and DISCOVER-seq.

2 - To be publishable, the paper needs to demonstrate that both methods can identify off-target sites that can be detected in cells. As a first problem, the overall transfection efficiency of the human fibroblast cells was low (~15%), which causes the very low editing frequency (5.69% for MMP14 and 9% for NEK1). The ideal sgRNA target site would have a high on-target site editing efficiency (>80%) and high specificity. However, with low on-target site activity, it is very likely that the mutation frequency at off-target sites would be very low or inexistent, although the sgRNA could still have a low or medium specificity that is misidentified in these experiments. It would be helpful if first the authors could optimize their transfection conditions aiming to increase the on-target site mutation rates and then next, evaluate the off-target sites predicted by SMRT-OTS and Nano-OTS. Additionally, it would be helpful if the authors could add the on-target site editing frequency in the results section.

3 - The authors did not show clear advantages of the long-read sequencing approach. Are there any sites that they found that cannot be sequenced or mapped by high-throughput short-read sequencing on the basis of whole genome sequencing data?

4 - The authors aimed to compare SMRT-OTS and Nano-OTS, however, the way that the comparison was performed might be improved. The samples for each method should be sampled to the same sequencing depth, allowing a fairly comparison. Indeed, correlation plots comparing both methods could be displayed for each target site alongside with the R2 value. Additionally, it would be great to see the technical reproducibility of both methods.

5 - The authors performed multiplex in vitro cleavage of the gDNA using three sgRNAs simultaneously. How would the authors ensure that the sensitivity of the assays is not decreased due to multiplex? It would be interesting to see data from experiments where sgRNAs were used also independently.

6 - It would add great value to the paper if the authors could evaluate more target sites in both independent and multiplex ways, at least 10 target sites, including very well characterized sgRNAs. By evaluating only three target sites, it is feasible to prove that both methods work, but is hard to derive any general conclusions. For example, with this very small set of off-targets, the authors found only one off-target site where the Cas9 in vitro cleavage activity was affected by the presence of a SNP. With a larger dataset more studies could be performed and would strength the conclusions.

Minor
1 - Throughout the text, the authors have confused the concepts of sgRNA:Cas9 binding and cleavage, stating binding where they mean cleavage.  sgRNA:Cas9 binding to the DNA does not mean that the DNA will be cut by the nuclease (Dagdas et al. A conformational checkpoint between DNA binding and cleavage by CRISPR-Cas9. Science Advances, 2017). It would clarify if the word "binding" was replaced by "cleavage".

2- Page 4 Lines 48-52. "The DNA is then repaired by non-homologous end joining (NHEJ), and small insertion and deletion mutations are typically introduced during this repair step". This sentence could be better elaborated, as the DNA is often, but not always repaired by NHEJ.

3 - Page 6 lines 32-34. The authors referred to the looped adapter-ligated DNA molecules as circular DNA. It would be more prudent to refer as covalently closed DNA molecules rather than circular DNA.

4 - The number of mismatches tolerated/considered for the analysis could be mentioned in the text.

5 - Page 5 lines 31-32.
a.     Authors should cite and discuss other relevant method not mentioned:
Cameron, P. et al. Mapping the genomic landscape of CRISPR-Cas9 cleavage. Nat. Methods 14, 600-606 (2017).

b.     Authors should also cite additional reference which update the most recent version of the method:
Lazzarotto, C. R. et al. Defining CRISPR-Cas9 genome-wide nuclease activities with CIRCLE-seq. Nat. Protoc. 13 (2018). doi:10.1038/s41596-018-0055-0

**Reviewer 2**

**Are you able to assess all statistics in the manuscript, including the appropriateness of statistical tests used?** Yes, and I have assessed the statistics in my report.

**Comments to author:**

In the manuscript, "Amplification-free long read sequencing reveals unforeseen CRISPR-Cas9 off-target activity in vitro", Höijer et al. describe  'Off-target sequencing" (OTS)  .. strategies using targeting sequencing to evaluate off-target cleavage by the CRISPR/Cas9 system. Though this work is interesting and potentially useful, there are a couple of key points where deeper analysis and work is needed to fully realize the potential.  I enumerate the following points :
General:
1. You continually use the term "binding sites", but I think these should more clearly be described as "cleavage sites". In order for the site to be detected by either of the assays being used, a DSB must occur.   The Cas9/guideRNA complex is likely binding to lots of other sites also without cleavage.
2. When you say CRISPR/Cas9 "provided the possibility to modify the DNA inside living cells", this implies that it was impossible without CRISPR/Cas9. But we were, of course, editing genes in cells prior to this system (most notably, the TALEN technology that preceded it). It has certainly streamlined and increased efficiency and ease of modifying DNA in living cells, but not introduced it
3. I'd also be careful in this background section to distinguish germline DNA editing from somatic. And by that I mean, there are not really ethical concerns with somatic editing
4. Figure 3B is hard to parse - presumably the authors are plotting only the overlap? It would also probably be helpful to split these out as separate facets instead of all in one graph, the multiple colored dots make it hard to understand.
De novo (HEK Genome reassembly):
5. Table S2 shows that the assembly you built has >2000 contigs. This seems less complete than hg38? The authors need to be more explicit about the value-added from their assembly. How does the DNA sequence differ that couldn't have been obtained from simple Illumina SNP calling? After your whole-genome HiFi sequencing, were any new features found that were not in the previous annotation?  
6. It's an overstatement to say that this "allowed us to detect the gRNA binding sites without any prior information about the human reference" - I'd suggest dialing this back to something like "we can detect gRNA binding sites even with a de novo assembled genome, allowing us to operate without use of a reference". But then statistics need to be clear on the operation comparing hg38 to your de novo reference.  I would especially point out that your (presumably) annotated the gene locations using existing reference information lifted over? And I would also _redesign_ your gRNA based on your assembly, not the hg38 designed gRNA.
7. Page 9, line 32 Did the HiFi data not unambiguously show a duplication in this region?  Supp Fig S1 looks to be slightly higher coverage - but running a CNV tool on this data shouldn't be too difficult?.
Method
8. The details on the methods (SMRT-OTS, Nano-OTS) are scant in the results - I would just _say_ (briefly) that the "block" is dephosphorylation for nano-ots and that you are capturing/enriching for SMRT-OTS.  Figure 1 is also small and hard to parse, I recommend increase font size and clarify especially in 1C. Futher - is the code used for analysis fully available? I see the "InSiDeR" package, but looking for the full code
9. It's not clear - but presumably each guideRNA was used individually to allow checking which off-target belongs to which guide?  This is not clear.
10. It seems important to look closely at the differences between off-target sites which agree between nano and smrt and ones which differ, and how they differ. The correlation, though "positive" in Figure 3B - is quite poor with most points on the axes (probably should report the correlation coefficient)? I expect these differences between methods to be artifacts between the methods of enrichment, but am curious how they differ. And why the intersection instead of the union - if it generates a pileup in either method - isn't it a potential off-target spot?  I'm especially curious about NEK1 where Pacbio didn't detect many, but nano did.
11. Only three guideRNAs used to assess?  I'm not at all convinced that these guideRNAs are representative of off-target activity. It seems that guides which are known to have strong
12. Is the argument for allele specific off-target (or on-target for that matter) that you need to have a good picture of the SNPs/haplotypes for a sample/genome in order to accurately edit it/design guides? But then I don't see that you need a reassembly for that - you could get that from just Illumina data? If there is another point here, the authors need to clarify.
13. This seems like a great opportunity to see if there are systematic differences between OTS sites that were predicted by a tool (eg  CHOP CHOP) and sites that were not predicted. If CHOP-CHOP (or other tools) allowed more mismatches, would these sites have been detected? What about the PAM mismatch (I found that language confusing.
14. So - 5 reads are enough to find a "real" off-target, but presumably there were many more reads that didn't show a pileup of an "off-target" site?  What fraction of reads resulted from Cas9 cleavage vs. just fragmentation?
15.  This section on searching for binding sites in "dark-matter" regions does not add substance to the paper. It is not convincing for me to draw conclusions either way - perhaps this is because the guides designed don't have off-taget in repetitive areas? A different guide design might help show the potential of this better.
In cell editing/off-targets
16. Transfection was effective in 10-15 % of how many cells? And it looks like the fraction of reads which showed mutations was very small from Fig 6B? Is this a matter of sequencing depth that nothing is observed at the off-target sites?  Could the authors at least have selected for successfully transfected cells, to look for cells which received the Cas9/guide?
17. Figure 6B :  are these indels relative to wildtype HEK (the assembly) or to Hg38? Why are there are so many indels outside of the Cas9 cleavage site?
18. If the point here is to show off-target - is it reasonable to do this in the context of in vitro DNA instead of DNA in the nucleus with the different chromatin configurations and conditions that would experience? 6C seems to suggest the "off-target" sites detected don't actually cause insertions or deletions? So what's the true utility?
19. Seems like a guide which causes _known_ off-targets in cells should have been used, to see if that could be predicted.  Or deeper sequencing was needed to see the off-targets (even if at low level).  As it stands, it seems that this method (with the 3 guides tested) doesn't really answer the off-target question it's designed to?

**Reviewer 3**

**Are you able to assess all statistics in the manuscript, including the appropriateness of statistical tests used?** There are no statistics in the manuscript.

**Comments to author:**

Reviewer comments for manuscript: Amplification-free long read sequencing reveals unforeseen CRISPR-Cas9 off-target activity in vitro (GBIO-D-20-00484)

The line numbers mentioned in the comments are the numbers on the left side of the page, although they don't match the lines in the text.

The authors proposed two long-read sequencing protocols (SMRT-OTS and Nano-OTS) for detection of CRISPR/Cas9 off-target sites in vitro. Both protocols are similar to previously proposed assays where CRISPR/Cas9 was used to perform targeted enrichment for SMART and nanopore sequencing. The authors applied these assays to detect off-target editing events. The authors tested the two protocols using genomic DNA of the HEK293 cell line and showed that they can detect some off-target sites that are not predicted by CHOPCHOP. They also found an allele-specific editing site caused by a heterozygous SNP. Finally, they tried to validate if the off-target events detected by the two in vitro assays exist in cell-based experiments. Unexpectedly, none of the off-target events were detected in cell-based experiments. The idea has some novelty and the manuscript is easy to understand, although there are some minor mistakes.


Major comments

My major issue with this manuscript is that the proposed methods lack comprehensive benchmarking. As shown in Figure 3, a large portion of gRNA sites detected from SMRT-OTS and Nano-OTS are inconsistent. There is no additional experiment to show which method is more accurate. A simple whole-genome sequencing of the edited DNA plus variant calling will give you a gold standard data set.

In addition, there is no evidence showing that the off-target sites detected in vitro actually exist in editing of live cells. As shown in Figure 6, there is no editing of the "off-target sites" in cell-based experiments.

I believe there should be some off-target sites in cell-based experiments. There is no evidence showing how many of the off-target sites in cell-based experiments can be detected in vitro using your protocols.


Minor comments

In method section, page 15, lines 1-2. "The ATXN10 gRNA (AUACAAAGGAUCAGAAUCCC) is used in our previous experiments on amplification-free PacBio sequencing of repeat expansions in the human genome 24, 31". Here you cited ref 24 which is a Nature Methods paper but I didn't find any overlapping author between ref 24 and the current manuscript. In addition, ref 24 is not related to PacBio sequencing and ATXN10. Please check that!

In the abstract (page 3, line 32). You claimed that you can detect gRNA binding sites without a reference genome. This claim doesn't make sense because you created a "reference genome" from high-quality whole-genome sequencing data and de novo assembly. In addition, you still need the reference genome to know which gene the off-target sites reside in.

In the abstract. "Finally, we performed CRISPR-Cas9 editing experiments and could find no evidence of off-target mutations in human fibroblasts" This sentence needs to be reorganized. For example, you can say "we performed CRISPR-Cas9 editing experiments in human fibroblasts but failed to find the off-target sites detected in vitro".

In page 4, first paragraph. CRISPR-Cas9 genome editing was not first demonstrated in 2013. It had been reported in 2012. (Jinek, M., et al. (2012). "A programmable dual-RNA-guided DNA endonuclease in adaptive bacterial immunity." Science 337(6096): 816-821.)

In page 6, line 54. "a single base pair resolution" should be "at single base pair resolution".

In page 8, line 10. "Due to a higher error rate in the Nano-OTS reads, the gRNA binding sites are sometimes predicted within a 10-20 bp interval instead of at exact base pair resolution." Nanopore reads have higher error rate than PacBio HiFi reads but you can perform error-correction before analysis.

As shown in Figure 2, the reads are highly biased in one side in MM14 and NEK1 sites. Any explanations?

In page 8, last line. In the in silico analysis of off-target sites, you only allowed up to three mismatch sites. 18 OTS-sites had at least four single nucleotide mismatches. How many of the 18 off-target sites can be predicted if four mismatch sites are allowed?

There is no description of code availability.

**Authors Response**

**Point-by-point responses to the reviewers’ comments:**

Reviewer #1: In "Amplification-free long read sequencing reveals unforeseen CRISPRCas9 off-target activity in vitro", Hoijer et al. described the development of SMRT-OTS and Nano-OTS, two orthogonal methods for identifying Cas9 genome-wide off-target activity in vitro, leveraging long-read sequencing platforms. The workflow for both methods is straightforward and use molecular biology strategies similar to CIRCLE-seq and SITE-seq for selective sequencing of Cas9 cleaved DNA molecules. However, the overall novelty of the methods and conclusions is unclear from the manuscript in its current form and, there a number of points that should be addressed to warrant publication in Genome Biology.

Major

1 - Although the strategies described appear straightforward, the authors did not perform any direct comparison with established methods by analyzing known benchmark sgRNAs targets such as VEGFA, RNF2 and EMX1, which makes it difficult to access the relative performance of SMRT-OTS and Nano-OTS. For completeness, target sites as the ones mentioned above could be evaluated for a fair comparison with published in vitro methods, such as CIRCLE-seq, Digenome-seq and SITE-seq. Indeed, if those targets could be evaluated, it would also be relevant to compare with GUIDE-seq and DISCOVER-seq.

*** Reply: We agree that the lack of benchmarking against other methods was a major weakness in the previous manuscript version, and have performed new experiments to address this issue. As suggested by the reviewer, SMRT-OTS and Nano-OTS have now been applied to the gRNAs EMX1, FANCF, RNF2 and VEGFA. These four gRNAs have all been used for benchmarking purposes in previous studies. The results from our OTS runs were compared against previous results for Digenome-seq, CIRCLE-seq and SITE-seq. 85% of the OTS-sites were detected by Digenome-seq and CIRCLEseq. This percentage is even higher if also comparing to SITE-seq, where data is available only for two of the gRNAs (VEGFA and EMX1). Thus, our results clearly show that our OTS-methods can detect the same sites as the other in vitro based approaches. The revised manuscript contains a new results section describing the method comparison, including a figure (Fig 5A) as well as supplementary figures and tables. We have also restructured the abstract to accommodate the new benchmarking results, and added a paragraph in the discussion to explain why there are some discrepancies between methods and highlight advantages and drawbacks. We believe that the method comparison significantly strengthened the manuscript and would like to thank this reviewer, as well as the other reviewers, for raising this important point. ***

2 - To be publishable, the paper needs to demonstrate that both methods can identify off-target sites that can be detected in cells. As a first problem, the overall transfection efficiency of the human fibroblast cells was low (~15%), which causes the very low editing frequency (5.69% for MMP14 and 9% for NEK1). The ideal sgRNA target site would have a high on-target site editing efficiency (>80%) and high specificity. However, with low on-target site activity, it is very likely that the mutation frequency at off-target sites would be very low or inexistent, although the sgRNA could still have a low or medium specificity that is misidentified in these experiments. It would be helpful if first the authors could optimize their transfection conditions aiming to increase the ontarget site mutation rates and then next, evaluate the off-target sites predicted by SMRT-OTS and Nano-OTS. Additionally, it would be helpful if the authors could add the on-target site editing frequency in the results section.

*** Reply: Yes, we agree that it is important to demonstrate that some of the detected off-targets can be identified also in cells. To address this issue, we have taken advantage of results from previous CRISPR-Cas9 experiments for the gRNAs EMX1 and VEGFA where off-targets were detected in cells. Off-target editing was confirmed at four of our predicted OTS-sites, with mutation levels varying between 1 to 25%. These four sites are now marked in Figure 5B and listed in the supplementary material. In our own editing experiments, we failed to identify off-target editing in cells, and as the reviewer suggests one possible explanation for this could be the low transfection rate or other experimental conditions. However, since off-target editing usually is unwanted we think that it is interesting to demonstrate that our experiments did not show any such effects. In the revised manuscript we show that CRISPR-Cas9 experiments either can give rise to off-target editing in cells (based on previous experiments on VEGFA and EMX1) or not (based on our own experiments on MMP14 and NEK1). We also discuss which experimental factors likely to affect the off-target editing efficiency in cells, and for this discussion we reference the SITE-seq paper where more detailed experiments were performed on this topic. ***

3 - The authors did not show clear advantages of the long-read sequencing approach. Are there any sites that they found that cannot be sequenced or mapped by highthroughput short-read sequencing on the basis of whole genome sequencing data?

*** Reply: This is yet another important point that has been raised by several of the reviewers. In the previous manuscript we did in fact find several larger editing rearrangements, which are more challenging or impossible to detect reliable with short read sequencing, but other than that we did not really demonstrate the advantage of long-read sequencing. Therefore, we have deliberately designed six gRNAs in “dark” regions of the genome, i.e. repetitive regions where short reads have difficulties to align. The selected targets were within the genes CRYAA, HSPA1A, IKBKG, OPN1LW, OTOA, and STRC and obtained from a recent study (Ebbert et al, Genome Biology 2019). Our results showed that all six on-target sites could be successfully recovered by Nano-OTS, despite having no coverage in in-house Illumina 30x WGS data. Also, several of the off-target sites were found to overlap with dark genic regions. We believe this new analysis provide strong evidence that long-read methods can detect on- and off-targets in regions of the genome that are difficult, or impossible, to study with short reads. In the manuscript we have included new text and figure panel (Figure 5C) on this analysis of dark genic regions in the results section. We also describe these new results in the abstract as well as in the discussion. The manuscript still contains a few sentences about the HEK293 de novo assembly that we used for our analysis instead of GRCh38, but since we believe the new analysis on dark regions is more interesting, we have played down the de novo assembly part and no longer mention it in the abstract or in the discussion. ***

4 - The authors aimed to compare SMRT-OTS and Nano-OTS, however, the way that the comparison was performed might be improved. The samples for each method should be sampled to the same sequencing depth, allowing a fairly comparison. Indeed, correlation plots comparing both methods could be displayed for each target site alongside with the R2 value. Additionally, it would be great to see the technical reproducibility of both methods.

*** Reply: We have now performed a comparison between SMRT-OTS and Nano-OTS after having downsampled the datasets so they contain the same number of aligned reads. Although this analysis did improve the overlap, there are still some discrepancies between the two methods. In the revised version, we discuss possible explanations for this, such as differences in Cas9 cleavage efficiency in the two protocols. Regarding technical reproducibility, we have not performed several replicate OTS-runs for the same gRNAs. The reason for this is that we rather wanted to focus on performing other experiments and testing more gRNAs. Also, we removed the previous Figure 3B showing the correlation between Nano-OTS and SMRT-OTS since several reviewers found this figure confusing. In our opinion that figure is not necessary in the revised manuscript since we now have other results that are more important and informative, such as the benchmarking against other sequencing-based methods for Cas9 cleavage site detection. ***

5 - The authors performed multiplex in vitro cleavage of the gDNA using three sgRNAs simultaneously. How would the authors ensure that the sensitivity of the assays is not decreased due to multiplex? It would be interesting to see data from experiments where sgRNAs were used also independently.

*** Reply: To address this comment, we performed a new experiment for EMX1, FANCF, RNF2 and VEGFA where the Cas9 cleavage was performed independently for each gRNA and made a comparison to a multiplexed run for the same four gRNAs. In the single-plex run we detected fewer sites than in the multiplexed run (75 vs 107) and 80% (n=60) of the single-plex sites were found also in the multiplexed pool. In our opinion, this is a good overlap. Since the multiplexed run in fact resulted in more detected sites, we can conclude that low level multiplexing did not have a negative impact on sensitivity in this experiment. We have now included a few of sentences about this experiment in the results section and also a supplementary figure (Fig S5) showing the overlap between single-plex vs multiplexed results. ***

6 - It would add great value to the paper if the authors could evaluate more target sites in both independent and multiplex ways, at least 10 target sites, including very well characterized sgRNAs. By evaluating only three target sites, it is feasible to prove that both methods work, but is hard to derive any general conclusions. For example, with this very small set of off-targets, the authors found only one off-target site where the Cas9 in vitro cleavage activity was affected by the presence of a SNP. With a larger dataset more studies could be performed and would strength the conclusions.

*** Reply: In the revised version of the manuscript, we have performed off-target sequencing for 10 additional gRNAs, both in multiplex and single-plex runs. Based on this data we have performed several new analyses proposed by the reviewers, such as analyses of the dark regions of the genome, additional comparisons SMRT-OTS vs Nano-OTS, benchmarking against other sequencing-based protocols (CIRCLE-seq, Digenome-seq, SITE-seq), and evaluation of multiplexing. We have deliberately chosen not to include all 13 gRNAs in all of our analyses, as it would be difficult to present in a clear and comprehensive way, and hope that the reviewers agree with how our data and results are presented. Importantly, our new results clearly show that our methods can be generalized also to other gRNAs and demonstrates the benefit of long-read information to investigate Cas9 cleavage in dark regions of the genome. We are thankful for this comment from Reviewer #1 and are confident that the manuscript has been considerably improved. ***

Minor 1 - Throughout the text, the authors have confused the concepts of sgRNA:Cas9 binding and cleavage, stating binding where they mean cleavage. sgRNA:Cas9 binding to the DNA does not mean that the DNA will be cut by the nuclease (Dagdas et al. A conformational checkpoint between DNA binding and cleavage by CRISPR-Cas9. Science Advances, 2017). It would clarify if the word "binding" was replaced by "cleavage".

*** Reply: We agree with this comment and have replaced ”gRNA binding” with ”Cas9 cleavage” or in some cases “Cas9 digestion” throughout the text. ***

2- Page 4 Lines 48-52. "The DNA is then repaired by non-homologous end joining (NHEJ), and small insertion and deletion mutations are typically introduced during this repair step". This sentence could be better elaborated, as the DNA is often, but not always repaired by NHEJ.

*** Reply: We understand Reviewer #1’s concern on how DNA is repaired post Cas9 digestion and have clarified this in the manuscript and also added a description of homology directed-repair (HDR). ***

3 - Page 6 lines 32-34. The authors referred to the looped adapter-ligated DNA molecules as circular DNA. It would be more prudent to refer as covalently closed DNA molecules rather than circular DNA.

*** Reply: Reviewer 1 is referring to the sentence ”…PacBio’s SMRT sequencing and produces highly accurate circular consensus reads…” The nomenclature defined by Pacific Biosciences for the sequencing of their SMRTbell sequencing libraries is circular consensus sequencing (CCS) (Travers et al. A flexible and efficient template format for circular consensus sequencing and SNP detection. Nucleic Acids Research, 2010), and hence the reads are called CCS reads. We have changed to the exact nomenclature in the manuscript. Although the molecules used to produce these kind of reads (the SMRTbell libraries) might be described better as “covalently closed DNA molecules” the correct definition of the read type is CCS reads. ***

4 - The number of mismatches tolerated/considered for the analysis could be mentioned in the text.

*** Reply: We use EMBOSS-Needle to align the gRNA sequences to the peaks, after which we apply a cut-off value (score at least 55) to get rid of those peaks that have little or no resemblance to any gRNA. With these settings we can tolerate at least 5 mismatches and also a few insertion/deletions. However, we do not see this filtering as an integral part of our methods and it is entirely possible to run OTS without doing any filtering at all. For those reasons, we don’t want to specify a number of tolerated mismatches in the text. Instead, we added a sentence in the results section to make the reader aware that specific analysis details for this study are available in the methods section. ***

5 - Page 5 lines 31-32. a. Authors should cite and discuss other relevant method not mentioned: Cameron, P. et al. Mapping the genomic landscape of CRISPR-Cas9 cleavage. Nat. Methods 14, 600-606 (2017).

*** Reply: Thanks for bringing our attention to this SITE-seq publication. It was very informative and has been cited in several places in the revised manuscript. ***

b. Authors should also cite additional reference which update the most recent version of the method: Lazzarotto, C. R. et al. Defining CRISPR-Cas9 genome-wide nuclease activities with CIRCLE-seq. Nat. Protoc. 13 (2018). doi:10.1038/s41596-018-0055-0

*** Reply: We agree that this is a relevant publication and it has now been cited. ***

Reviewer #2: In the manuscript, "Amplification-free long read sequencing reveals unforeseen CRISPR-Cas9 off-target activity in vitro", Höijer et al. describe 'Off-target sequencing" (OTS) .. strategies using targeting sequencing to evaluate off-target cleavage by the CRISPR/Cas9 system. Though this work is interesting and potentially useful, there are a couple of key points where deeper analysis and work is needed to fully realize the potential. I enumerate the following points :

General:

1. You continually use the term "binding sites", but I think these should more clearly be described as "cleavage sites". In order for the site to be detected by either of the assays being used, a DSB must occur. The Cas9/guideRNA complex is likely binding to lots of other sites also without cleavage.

*** Reply: This point was also raised by Reviewer #1 (minor point 1). It has been addressed by replacing “gRNA binding” with “Cas9 cleavage” or “Cas9 digestion” throughout the manuscript. ***

2. When you say CRISPR/Cas9 "provided the possibility to modify the DNA inside living cells", this implies that it was impossible without CRISPR/Cas9. But we were, of course, editing genes in cells prior to this system (most notably, the TALEN technology that preceded it). It has certainly streamlined and increased efficiency and ease of modifying DNA in living cells, but not introduced it

*** Reply: Thank you for pointing this out. We have now changed this sentence and even borrowed some of the words from the above comment. It now reads: “The CRISPR-Cas9 system is one of the most important breakthroughs in modern biotechnology, as it has increased the efficiency and ease of modifying DNA in living cells”. ***

3. I'd also be careful in this background section to distinguish germline DNA editing from somatic. And by that I mean, there are not really ethical concerns with somatic editing

*** Reply: This is a good point and we have now changed the sentence in the introduction to the following: “CRISPR-Cas9 also shows great promise for clinical use, even though the ethical aspects of human germline genome editing require careful consideration. ***

4. Figure 3B is hard to parse - presumably the authors are plotting only the overlap? It would also probably be helpful to split these out as separate facets instead of all in one graph, the multiple colored dots make it hard to understand.

*** Reply: We removed Figure 3B since several reviewers found it uninformative. In the revised version we have instead focused on other benchmarking analyses to evaluate the performance of SMRT-OTS and Nano-OTS (see answer to Reviewer #1, major point 1). ***

De novo (HEK Genome reassembly):

5. Table S2 shows that the assembly you built has >2000 contigs. This seems less complete than hg38? The authors need to be more explicit about the value-added from their assembly. How does the DNA sequence differ that couldn't have been obtained from simple Illumina SNP calling? After your whole-genome HiFi sequencing, were any new features found that were not in the previous annotation?

*** Reply: We have toned down the results of the de novo assembly and instead highlight the results of the new experiments we performed to identify Cas9 cleavage in dark regions of the genome where short reads fail to align (see also answer to Reviewer #1, major point 3) ***

6. It's an overstatement to say that this "allowed us to detect the gRNA binding sites without any prior information about the human reference" - I'd suggest dialing this back to something like "we can detect gRNA binding sites even with a de novo assembled genome, allowing us to operate without use of a reference". But then statistics need to be clear on the operation comparing hg38 to your de novo reference. I would especially point out that your (presumably) annotated the gene locations using existing reference information lifted over? And I would also _redesign_ your gRNA based on your assembly, not the hg38 designed gRNA.

*** Reply: We agree that this probably was an overstatement. We have rephrased this sentence in the discussion, and no longer mention the de novo assembly results in the abstract. ***

7. Page 9, line 32 Did the HiFi data not unambiguously show a duplication in this region? Supp Fig S1 looks to be slightly higher coverage - but running a CNV tool on this data shouldn't be too difficult?.

*** Reply: This is a good point and we have now performed SV analysis on the HiFi data. However, the SV analysis not detect any CNVs or duplications in this region. This means that we at this point we have no unambiguous evidence of a duplication in this region. However, since the presence/absence of a duplication is not of any direct relevance for our results or conclusions, we don’t feel the need to investigate further. In the revised manuscript we now mention that the SV analysis failed to detect duplications in the region and also include the VCF file with HiFi SV data as a supplementary data file. ***

Method 8. The details on the methods (SMRT-OTS, Nano-OTS) are scant in the results - I would just _say_ (briefly) that the "block" is dephosphorylation for nano-ots and that you are capturing/enriching for SMRT-OTS. Figure 1 is also small and hard to parse, I recommend increase font size and clarify especially in 1C. Futher - is the code used for analysis fully available? I see the "InSiDeR" package, but looking for the full code

*** Reply: We have now added more details to the description of the methods in Figure 1 and its legend. We also increased the font size. Moreover, we will make detailed lab protocols available from protocols.io. The Nano-OTS protocol is already online (https://www.protocols.io/view/nano-ots-bjmukk6w) and the SMRT-OTS protocol will be published shortly. Regarding the software, the Insider github page has been updated. The code and analysis steps and code, including an example dataset for test runs, are available from the following URL: https://github.com/UppsalaGenomeCenter/InSiDeR. ***

9. It's not clear - but presumably each guideRNA was used individually to allow checking which off-target belongs to which guide? This is not clear.

*** Reply: No, we have not performed any runs with each gRNA individually. Although it would be entirely possible to use one single gRNA per sequencing run, this would be a rather costly and inefficient approach unless libraries are barcoded (and we have not attempted barcoding yet). Instead, we use the sequence information in each peak to determine which gRNA is likely to bind there. We have now clarified this in the revised manuscript. ***

10. It seems important to look closely at the differences between off-target sites which agree between nano and smrt and ones which differ, and how they differ. The correlation, though "positive" in Figure 3B - is quite poor with most points on the axes (probably should report the correlation coefficient)? I expect these differences between methods to be artifacts between the methods of enrichment, but am curious how they differ. And why the intersection instead of the union - if it generates a pileup in either method - isn't it a potential off-target spot? I'm especially curious about NEK1 where Pacbio didn't detect many, but nano did.

*** Reply: This is an important point, and we have now performed a more comprehensive comparison not only between Nano-OTS and SMRT-OTS, but also with other in vitro based methods (Digenome-seq, CIRCLE-seq and SITE-seq). Our conclusions are that there is rather good overlap between our OTS and the other in vitro methods. Also, there are a number of sites that are found only by one of the methods. Partly these could be explained by artefacts, as the reviewer suggests. But we believe that many of them are due to experimental differences, such as Cas9 concentrations. In support of this hypothesis, the SITE-seq article showed that many more sites were detected when higher Cas9 concentrations were used. In the revised manuscript we discuss the method differences in more detail. However, we have chosen not to look in detail at the differences in gRNA binding sequences between the different methods since we don’t think such an analysis would benefit our manuscript (see also answer to Reviewer #2, point 13 below). Regarding Figure 3B, it has now been removed since all reviewers found it confusing. Finally, we chose to use the intersection of Nano-OTS and SMRT-OTS when generating a high confidence set of Cas9 cleavage sites for ATXN10, MMP14 and NEK1. In our new benchmarking analysis, we have instead used the union in our analysis, as suggested above. ***

11. Only three guideRNAs used to assess? I'm not at all convinced that these guideRNAs are representative of off-target activity. It seems that guides which are known to have strong

*** Reply: We agree the low number of gRNAs was a weakness, and we have therefore included 10 additional gRNAs in the revised manuscript. Among these are EMX1, FANCF, RNF2 and VEGFA which have been used to assess other in vitrobased sequencing methods for off-target analysis. Also, we included other gRNAs design to bind “dark” regions of the human genome. ***

12. Is the argument for allele specific off-target (or on-target for that matter) that you need to have a good picture of the SNPs/haplotypes for a sample/genome in order to accurately edit it/design guides? But then I don't see that you need a reassembly for that - you could get that from just Illumina data? If there is another point here, the authors need to clarify.

*** Reply: It is correct that allele specific binding could have been detected also by Illumina data, and that de novo assembly is not required for this purpose. However, we have chosen to perform HiFi sequencing on PacBio Sequel II since that is currently the best approach to get detailed information of SNPs/haplotypes in the genome. In the revised manuscript we have toned down the part on de novo assembly, and clarified in the text. ***

13. This seems like a great opportunity to see if there are systematic differences between OTS sites that were predicted by a tool (eg CHOP CHOP) and sites that were not predicted. If CHOP-CHOP (or other tools) allowed more mismatches, would these sites have been detected? What about the PAM mismatch (I found that language confusing.

*** Reply: This is an interesting remark and we would like to thank Reviewer #2 for this proposal. However, we have chosen a different focus in our manuscript and adding these types of analyses would, not only lengthen the manuscript considerably, but also make the storyline too inconsistent. This said, all data needed to make a comparative analysis of OTS-sites in relation to in silico predicted sites are now available in the supplementary material for anyone who would like to investigate this in detail. ***

14. So - 5 reads are enough to find a "real" off-target, but presumably there were many more reads that didn't show a pileup of an "off-target" site? What fraction of reads resulted from Cas9 cleavage vs. just fragmentation?

*** Reply: In both SMRT-OTS and Nano-OTS there are a number of background reads that are not a result of Cas9 cleavage. To ensure we do not include these reads in our OTS results, when molecules are randomly fragmented at the same sites and form similar mapping patterns as our OTS reads, we screen all OTS peaks for gRNA similarity by performing a pairwise sequence alignment. Peaks that do not meet our criteria are filtered out in this step. Optionally, our analysis can be performed without this filtering. We have now clarified this analysis step in the results section. ***

15. This section on searching for binding sites in "dark-matter" regions does not add substance to the paper. It is not convincing for me to draw conclusions either way - perhaps this is because the guides designed don't have off-taget in repetitive areas? A different guide design might help show the potential of this better.

*** Reply: We would like to thank the reviewer for this comment as it gave us inspiration to design a new experiment to demonstrate the value of long reads in our OTS protocols. For more details, please see answer to Reviewer #1, major point 3. ***

In cell editing/off-targets

16. Transfection was effective in 10-15 % of how many cells? And it looks like the fraction of reads which showed mutations was very small from Fig 6B? Is this a matter of sequencing depth that nothing is observed at the off-target sites? Could the authors at least have selected for successfully transfected cells, to look for cells which received the Cas9/guide?

*** Reply: In the methods section it is mentioned that 6 x 105 cells were used for the transfection experiments. Of these, transfection was effective in 10-15% of cells. From our sequencing reads, we can estimate the on-target editing efficiency to 32-48% for MMP14 and 58-87% for NEK1. These numbers were also requested by Reviewer #1 and are now mentioned in the results section. We believe that these numbers are sufficiently high to study editing events at the Cas9 cleavage sites. In theory, we could have selected only for successfully transfected cells by first performing a FACS sorting followed by cell culturing. This was something we wanted to avoid since FACS sorting is a harsh method for the cells and can result in cell death. Moreover, our aim was to study the different editing events in an unbiased cell population that has not been subjected to selection. Cell culture post editing can lead to a growth advantage for cells with different genetic edits, taking over the cell population. ***

17. Figure 6B : are these indels relative to wildtype HEK (the assembly) or to Hg38? Why are there are so many indels outside of the Cas9 cleavage site?

*** Reply: Our CRISPR-Cas9 genome editing experiments were performed in fibroblast cells and not in HEK293 cells. Since the fibroblast DNA has not been whole genome sequenced, the indels in Figure 6B are relative to GRCh38. It is difficult to know why there are indels outside of the Cas9 cleavage site, but we suspect that many of them are introduced during the PCR amplification, in particular in homopolymer regions where the polymerase is prone to introducing indel errors. This is the reason why we have also performed re-sequencing of control fibroblast DNA. We can use the control data to correct for PCR artefacts. ***

18. If the point here is to show off-target - is it reasonable to do this in the context of in vitro DNA instead of DNA in the nucleus with the different chromatin configurations and conditions that would experience? 6C seems to suggest the "off-target" sites detected don't actually cause insertions or deletions? So what's the true utility?

*** Reply: Yes, in many cases it is desirable to detect off-target editing after the CRISPR-Cas9 experiments have been performed and methods like GUIDE-seq and DISCOVER-seq can be used for this purpose. However, such methods are laborious and time consuming since they require genome editing to be performed. Our in vitro methods are much easier and faster. In particular the Nano-OTS approach that can be performed in 1-2 days on a cheap portable sequencing instrument. Moreover, our new experiments demonstrate that some of the identified Cas9 cleavage sites are indeed edited in cells (answer to Reviewer #1, major point 2) so our methods can clearly be used for that purpose. Also, it can sometimes be of more interest to study Cas9 cleavage in vitro, rather than performing experiments on cells. For example, when designing gRNAs for sequence capture experiments, when studying allele specific gRNA binding, or when screening large panels of gRNAs to identify potential candidates for cellular experiments. ***

19. Seems like a guide which causes _known_ off-targets in cells should have been used, to see if that could be predicted. Or deeper sequencing was needed to see the off-targets (even if at low level). As it stands, it seems that this method (with the 3 guides tested) doesn't really answer the off-target question it's designed to?

*** Reply: These points have also been raised by the other reviewers. To address this, new experiments have been performed and results for 13 different gRNAs are included in the revised version. See answers to Reviewer #1 above (major points 1,3 & 6). ***

Reviewer #3: Reviewer comments for manuscript: Amplification-free long read sequencing reveals unforeseen CRISPR-Cas9 off-target activity in vitro (GBIO-D-20- 00484)

The line numbers mentioned in the comments are the numbers on the left side of the page, although they don't match the lines in the text.

The authors proposed two long-read sequencing protocols (SMRT-OTS and NanoOTS) for detection of CRISPR/Cas9 off-target sites in vitro. Both protocols are similar to previously proposed assays where CRISPR/Cas9 was used to perform targeted enrichment for SMART and nanopore sequencing. The authors applied these assays to detect off-target editing events. The authors tested the two protocols using genomic DNA of the HEK293 cell line and showed that they can detect some off-target sites that are not predicted by CHOPCHOP. They also found an allele-specific editing site caused by a heterozygous SNP. Finally, they tried to validate if the off-target events detected by the two in vitro assays exist in cell-based experiments. Unexpectedly, none of the off-target events were detected in cell-based experiments. The idea has some novelty and the manuscript is easy to understand, although there are some minor mistakes.

Major comments

My major issue with this manuscript is that the proposed methods lack comprehensive benchmarking. As shown in Figure 3, a large portion of gRNA sites detected from SMRT-OTS and Nano-OTS are inconsistent. There is no additional experiment to show which method is more accurate. A simple whole-genome sequencing of the edited DNA plus variant calling will give you a gold standard data set.

*** Reply: Thank you for this important comment. The lack of benchmarking is something that has been pointed out by all reviewers and we have now addressed this with new experiments and comparisons to Digenome-Seq, CIRCLE-seq and SITE-seq (see answer to Reviewer #1, major point 1). Our results show that both Nano-OTS and SMRT-OTS results have good overlap with Cas9 cleavage sites detected by the other methods, suggesting a low percentage of false positives. However, we see that experimental factors such as Cas9 concentration and read depth can have a significant impact on the number identified sites and this can explain some of the discrepancies between the methods. Here it should be noted that there is substantial variation in results also for the Digenome-Seq, CIRCLE-seq and SITE-seq methods, and not only for Nano-OTS and SMRT-OTS. Regarding the suggestion to perform WGS of edited DNA, we don’t think this is a good way to evaluate the methods. We have strong reason to believe that many of the sites that are detected in vitro will not give rise to genome editing in cells (see also answer to Reviewer #1, major point 2) ***

In addition, there is no evidence showing that the off-target sites detected in vitro actually exist in editing of live cells. As shown in Figure 6, there is no editing of the "offtarget sites" in cell-based experiments.

*** Reply: This has been addressed by new experiments. See answer to Reviewer #1, major point 2 above. ***

I believe there should be some off-target sites in cell-based experiments. There is no evidence showing how many of the off-target sites in cell-based experiments can be detected in vitro using your protocols.

*** Reply: We agree that the absence of off-target editing in our cell experiments is unexpected. However, we believe there are many other factors that could be important, such as the CRISPR-Cas9 protocol used for editing, the chromatin conformation, DNA repair system in the cells etc. Our new experiments show that some of the OTS-sites for EMX1 and VEGFA indeed are edited in cells (see answer to Reviewer #1, major point 2). However, to properly answer this question we would need to perform a new round of genome editing experiments, ideally to look at in vivo editing. However, we believe this is outside the scope of this initial study where mainly focus on presenting our new OTS-methods. ***

Minor comments In method section, page 15, lines 1-2. "The ATXN10 gRNA (AUACAAAGGAUCAGAAUCCC) is used in our previous experiments on amplificationfree PacBio sequencing of repeat expansions in the human genome 24, 31". Here you cited ref 24 which is a Nature Methods paper but I didn't find any overlapping author between ref 24 and the current manuscript. In addition, ref 24 is not related to PacBio sequencing and ATXN10. Please check that!

*** Reply: Many thanks. This was an error and it has now been corrected. ***

In the abstract (page 3, line 32). You claimed that you can detect gRNA binding sites without a reference genome. This claim doesn't make sense because you created a "reference genome" from high-quality whole-genome sequencing data and de novo assembly. In addition, you still need the reference genome to know which gene the offtarget sites reside in.

*** Reply: As mentioned also in the answers to Reviewer #2, we agree that this was an overstatement. We have rephrased this sentence in the discussion, and no longer mention the de novo analysis in the abstract. Overall, we have toned down the results of the de novo assembly and instead focus on our new experiments to identify Cas9 cleavage in dark regions of the genome. ***

In the abstract. "Finally, we performed CRISPR-Cas9 editing experiments and could find no evidence of off-target mutations in human fibroblasts" This sentence needs to be reorganized. For example, you can say "we performed CRISPR-Cas9 editing experiments in human fibroblasts but failed to find the off-target sites detected in vitro".

*** Reply: This sentence in the abstract has been reorganized and now reads: “Even though SMRT-OTS and Nano-OTS identified several sites with previously validated offtarget editing activity in cells, our own CRISPR-Cas9 editing experiments in human fibroblasts did not give rise to detectable off-target mutations at the in vitro-predicted sites.” ***

In page 4, first paragraph. CRISPR-Cas9 genome editing was not first demonstrated in 2013. It had been reported in 2012. (Jinek, M., et al. (2012). "A programmable dualRNA-guided DNA endonuclease in adaptive bacterial immunity." Science 337(6096): 816-821.)

*** Reply: It is correct that Jinek et al demonstrated that reprogrammed RNPs could target novel sequences. This was demonstrated targeting plasmids, but it is debatable if this can be referred to as genome editing (Christin JR, Beckert MV. Origins and Applications of CRISPR-Mediated Genome Editing, Einstein J Biol Med, 2016; www.broadinstitute.org/what-broad/areas-focus/project-spotlight/crispr-timeline). However, to address Reviewer #3’s comment and to clarify in the manuscript we have changed the sentence describing this to “CRISPR-Cas9 genome editing in eukaryotic cells was first demonstrated in 2013…” ***

In page 6, line 54. "a single base pair resolution" should be "at single base pair resolution".

*** Reply: Ok, fixed ***

In page 8, line 10. "Due to a higher error rate in the Nano-OTS reads, the gRNA binding sites are sometimes predicted within a 10-20 bp interval instead of at exact base pair resolution." Nanopore reads have higher error rate than PacBio HiFi reads but you can perform error-correction before analysis.

*** Reply: We agree that this was described a bit crudely. What we actually see is that the nanopore reads do not all start/end at the exact same position as is the case for CCS reads. This is due to how the ONT sequencing and primary analysis work, rather than that the reads have a higher error rate than CCS reads. We have changed this sentence in the manuscript to clarify. ***

As shown in Figure 2, the reads are highly biased in one side in MM14 and NEK1 sites. Any explanations?

*** Reply: Yes, this is due to the orientation of the gRNA-Cas9 complex relative to the target sequence. After Cas9 cleavage the complex is still bound to the DNA of the 5’- side of the gRNA and this will make adapter ligation less efficient this DNA molecule. To clarify this to the readers we have explained this in the figure legend of Figure 2. ***

In page 8, last line. In the in silico analysis of off-target sites, you only allowed up to three mismatch sites. 18 OTS-sites had at least four single nucleotide mismatches. How many of the 18 off-target sites can be predicted if four mismatch sites are allowed?

*** Reply: 13 of the 18 OTS-sites would have been identified by CHOPCHOP by allowing four mismatches. But at the same time, CHOPCHOP would likely have predicted a very large number of false positive sites if four mismatches were allowed. In fact, it is not possible to set a value higher than three for the maximum allowed mismatches for CHOPCHOP, at least not in the web version that we have been running (https://chopchop.cbu.uib.no), indicating that an analysis with more mismatches would not be useful. ***

There is no description of code availability.

*** Reply: The analysis tool Insider for detection of OTS peaks is available from GitHub: https://github.com/UppsalaGenomeCenter/InSiDeR. This link is now included in the “Availability of data and materials” section. ***

**Second round of review**

**Reviewer 1**

Although the revised version looks better than the original version, there are still key questions that remain to be answered:

1 - What proportion of validated GUIDE-seq/DISCOVER-seq/CIRCLE-seq/CHANGE-seq/Digenome-seq off-target sites is detected by these methods in the sites evaluated? What is the sensitivity of these methods? Are they able to identify in vitro sites of bona fide cellular off-target activity?

2 - Previous methods (such CIRCLE-seq, Digenome-seq, GUIDE-seq, CHANGE-seq, SITE-seq) characterized at least 8-10 sgRNAs target sites (well known and benchmarked). For this work to be publishable more target sites need to be characterized in vitro and validated in a cell-based context. It would be useful to see more of these data, to understand the sensitivity of these two new methods.

3 - Can these methods reveal any useful off-target sites that cannot be detected by the existing methods? Are the off-target sites identified in the 'dark genomic' region by Nano-OTS bona fide cellular off-targets? What are the strengths of these long-read approaches for identification of Cas9 genome-wide activity?

4 - The session "A single nucleotide polymorphism can induce allele-specific Cas9 cleavage" needs more data. The authors found only one off-target site where the Cas9 in vitro cleavage activity was affected by the presence of a SNP.

**Reviewer 2**

The authors have done a lot of the requested work - though I would still have liked to have seen how the off-target measurement performs via bioinformatic tools like CHOP-CHOP (Rev2 Point 13) - I am satisfied with their response.

**Reviewer 3**

Most of my major and minor comments are addressed by the authors.

I understand the reluctance to perform genome-wide in vivo assay to compare results with cell-based experiments. I think it is fine that they are unable to address this point.

For code availability: The repository https://github.com/UppsalaGenomeCenter/InSiDeR is a little too rough with limited explanations. Ideally it should be self-evident even when a user does not read the original paper, to know what this tool does, how it does it, and what are the expected results and how to change arguments based on sequencing depth, etc.

**Authors Response**

**Point-by-point responses to the reviewers’ comments:**

Reviewer #1: Although the revised version looks better than the original version, there are still key questions that remain to be answered:

1 - What proportion of validated GUIDE-seq/DISCOVER-seq/CIRCLE-seq/CHANGEseq/Digenome-seq off-target sites is detected by these methods in the sites evaluated? What is the sensitivity of these methods? Are they able to identify in vitro sites of bona fide cellular off-target activity?

*** Reply: We thank the reviewer for this comment. CHANGE-seq is a recent publication that we have read with great interest and included among our references. However, we believe that the questions above have already been addressed in the current version of the manuscript. When it comes to validated off-targets, we have examined sites detected in cells both in the CIRCLE-seq and Digenome-seq studies and having an allele frequency of at least 1%. There were four off-target sites that met these criteria. All four of these sites (100%) were detected by Nano-OTS. However, SMRT-OTS only detected two of these sites (50%). It can be argued that we should rather have chosen to include results from other studies, or to select other cut-offs for allele frequencies, but we don’t really see the point of doing this. The main point here was to determine that our methods indeed can identify off-target sites that induce mutations in cells, and this has now been done. Regarding sensitivity, this is a complicated issue since there are many experimental factors that influence how many off-targets are detected. For example, the concentration of Cas9 enzyme is strongly correlated with the amount of off-targets detected in in vitro experiments. This was clearly shown in the SITE-seq publication where experiments were performed using different Cas9 concentrations. In addition to Cas9 concentrations, there might be other experimental factors that could influence the results. For this reason, there is no obvious way to create a “truth set” of off-target sites that are expected to detected for a specific gRNA. What we have done instead is to compare our OTS results to off targets detected by Digenome-seq, CIRCLE-seq and SITE-seq. Our results show that there is good concordance between our long-read OTS methods and the short-read protocols, in particular for the strong off-target sites. However, we don’t think it is possible at this point to put an exact number of the sensitivity, not only for the OTSmethods but for any method to detect Cas9 cleavage in vitro. ***

2 - Previous methods (such CIRCLE-seq, Digenome-seq, GUIDE-seq, CHANGE-seq, SITE-seq) characterized at least 8-10 sgRNAs target sites (well known and benchmarked). For this work to be publishable more target sites need to be characterized in vitro and validated in a cell-based context. It would be useful to see more of these data, to understand the sensitivity of these two new methods.

*** Reply: In this study we have characterized 13 gRNAs. Of these, 4 gRNAs (VEGFA, EMX1, FANCF and VEGFA) have been used in several of the studies mentioned above. All of the expected on-target sites were detected by Nano-OTS and SMRTOTS. In addition, 85% of the Cas9 cleavage sites found by our OTS-methods were detected also by Digenome-seq, CIRCLE-seq or SITE-seq. We believe that this is sufficient for benchmarking purposes and don’t think experiments using additional gRNAs would add anything to this study. We want to emphasize that the aim of our study is not only to benchmark against previous studies, but also to examine advantages with the long-read protocols, such as studying Cas9 cleavage in dark genomic regions. For that reason, we have selected to included several new gRNAs in this study that have not been used previously. We hope that the reviewer can understand our reasoning when it comes to the gRNAs used in this study. ***

3 - Can these methods reveal any useful off-target sites that cannot be detected by the existing methods? Are the off-target sites identified in the 'dark genomic' region by Nano-OTS bona fide cellular off-targets? What are the strengths of these long-read approaches for identification of Cas9 genome-wide activity?

*** Reply: These are important questions. Since many of these dark genomic regions are located in medically relevant genes, it is likely that unwanted off-target mutations in those regions could have negative functional consequences. Although it would be very interesting to investigate this in detail, we believe functional characterization of offtarget mutations is outside the scope of the present study. However, we think our OTSmethods are valuable since they for the first time enable researchers to detect offtargets also in these complex regions of the genome. In addition, our OTS-methods have the advantage of being amplification-free. This should remove potential biases in the library preparation and also preserve epigenetic signals in the DNA, which could be of interest for example when studying the effect of DNA methylation on Cas9 cleavage efficiency. Finally, the Nano-OTS protocol is a fast and simple method that can be run on a portable sequencing instrument, thereby making it easily accessible for researchers without the need of submitting samples to a sequencing core lab. In summary, we are confident that our methods have several advantages. That is why we want to make them available. But ultimately it will be up to each individual researcher to decide on what method to use in each specific case. ***

4 - The session "A single nucleotide polymorphism can induce allele-specific Cas9 cleavage" needs more data. The authors found only one off-target site where the Cas9 in vitro cleavage activity was affected by the presence of a SNP.

*** Reply: We have now performed this analysis based on all seven SMRT-OTS datasets instead of only three as in the previous manuscript version. However, we did not find any additional examples of allele-specific Cas9 cleavage, suggesting that such events are relatively rare. We have moved the section on allele-specific Cas9 cleavage so that the result sections follow a logical order and also made some minor changes to the text. ***

Reviewer #2: The authors have done a lot of the requested work - though I would still have liked to have seen how the off-target measurement performs via bioinformatic tools like CHOP-CHOP (Rev2 Point 13) - I am satisfied with their response.

*** Reply: Ok, many thanks for this comment. ***

Reviewer #3: Most of my major and minor comments are addressed by the authors. I understand the reluctance to perform genome-wide in vivo assay to compare results with cell-based experiments. I think it is fine that they are unable to address this point.

For code availability: The repository https://github.com/UppsalaGenomeCenter/InSiDeR is a little too rough with limited explanations. Ideally it should be self-evident even when a user does not read the original paper, to know what this tool does, how it does it, and what are the expected results and how to change arguments based on sequencing depth, etc.

*** Reply: Thank you. Regarding the GitHub repository, we will continue to work on it and make it more user friendly. ***
